# Molecular characterisation of cell line models for triple-negative breast cancers

**DOI:** 10.1186/1471-2164-13-619

**Published:** 2012-11-14

**Authors:** Anita Grigoriadis, Alan Mackay, Elodie Noel, Pei Jun Wu, Rachel Natrajan, Jessica Frankum, Jorge S Reis-Filho, Andrew Tutt

**Affiliations:** 1 Breakthrough Breast Cancer Research Unit, Guy’s Hospital, King’s Health Partners AHSC, King’s College London School of Medicine, London, SE1 9RT, UK; 2 The Breakthrough Breast Cancer Research Centre, The Institute of Cancer Research, London, UK; 3 Current affiliation: Department of Pathology and Human Oncology and Pathogenesis Program, Memorial Sloan-Kettering Cancer Center, New York, NY, 10065, USA

**Keywords:** Microarray, Gene expression profiling, Comparative genomic hybridisation, Methylation arrays, Triple negative, Breast cancer

## Abstract

**Background:**

Triple-negative breast cancers (BC) represent a heterogeneous subtype of BCs, generally associated with an aggressive clinical course and where targeted therapies are currently limited. Target validation studies for all BC subtypes have largely employed established BC cell lines, which have proven to be effective tools for drug discovery.

**Results:**

Given the lines of evidence suggesting that BC cell lines are effective tools for drug discovery, we assessed the similarities between triple-negative BCs and cell lines, to identify *in vitro* representatives, modelling the diversity within this BC subtype. 25 BC cell lines, enriched for those lacking ER, PR and HER2 expression, were subjected to transcriptomic, genomic and epigenomic profiling analyses and comparisons were made to existing knowledge of corresponding perturbations in triple-negative BCs. Transcriptional analysis segregated ER-negative BC cell lines into three groups, displaying distinctive abundances for genes involved in epithelial-mesenchymal transition, apocrine and high-grade carcinomas. DNA copy number aberrations of triple-negative BCs were well represented in cell lines and genes with coordinately altered gene expression showed similar patterns in tumours and cell lines. Methylation events in triple-negative BCs were mostly retained in epigenomes of cell lines. Combined methylation and gene expression analyses revealed a subset of genes characteristic of the Claudin-low BC subtype, exhibiting epigenetic-regulated gene expression in BC cell lines and tumours, suggesting that methylation patterns are likely to underpin subtype-specificity.

**Conclusion:**

Here, we provide a comprehensive analysis of triple-negative BC features on several molecular levels in BC cell lines, thereby creating an in-depth resource to access the suitability of individual lines as experimental models for studying BC tumour biology, biomarkers and possible therapeutic targets in the context of preclinical target validation.

## Background

Oestrogen-receptor (ER) negative breast cancer (BC) accounts for approximately 20% of all newly diagnosed breast malignancies [1-3]. Clinically, however, this group of BCs contains different subtypes and can be subdivided into either HER2-positive or triple-negative BCs, defined by very low or absent immunohistochemical expression of ER and progesterone receptor (PR), and low expression and lack of amplification of HER2 [4]. Triple-negative BCs account for 10-15% of all breast tumours and are mostly of high grade, have a high incidence of *TP53* mutations, and show proliferative characteristics with a higher propensity to spread to visceral organs [4]. Sharing many of these phenotypic features with triple-negative BCs are breast tumours of the ‘intrinsic’ basal-like subtype. These tumours generally lack ER and HER2 expression and are molecularly characterised by the expression of genes associated with both basal epithelium and myoepithelium of the normal mammary gland (*e.g*. *KRT5/6*, *KRT14, VIM, CDH3, CRYAB, CAV1* and *CAV2,* as well as *EGFR*) [2,5]. Approximately, 80% of triple-negative BCs show features of basal-like BCs [4,6,7]. While most triple-negative BCs show aggressive clinical behaviour and have very limited targeted therapies, they also encompass subgroups of cancers sensitive to chemotherapy and having a good prognosis [4]. Hence, continuous efforts to characterise this BC population have already identified several subgroups. One of the proposed groups comprise “Claudin-low” tumours, which are characterised by gene expression profiles similar to those found in the so-called breast ‘cancer stem cell’ populations [8], while other subgroups were classified as having higher expression of the interferon-related or apocrine genes [9-12]. BC cell lines are essential tools in BC research and have been widely used to elucidate BC biology and new therapies [13,14]. Since cell lines are easily propagated and genetically manipulated, extensive information about their transcriptome, genome and to a lesser extent epigenome has been produced [11,15-19]. Several studies have compared and integrated gene expression profiles and genomic alterations between primary breast tumours and BC cell lines, demonstrating that the heterogeneity found in primary BCs is to a certain extent recapitulated in the panel of commonly used BC cell lines [15,16,18]. Given the increasing knowledge of the diversity and complexity among BC subtypes it has also become evident that no individual cell line will recapitulate all aspects of the disease. Here we interrogated genome-wide transcriptional profiles with genomic and epigenetic profiling in a collection of 25 BC cell lines enriched for those of triple-negative phenotype. We have focused on gene signatures, underlying DNA copy number aberrations (CNAs) and epigenetic events specifically associated with triple-negative BCs. By cataloguing these perturbations on a gene-centric basis we have extended the characterisation of these BC cell lines and offer valuable insights on their suitability in modelling certain features of this heterogeneous disease.

## Results

### BC cell lines segregate into three groups based on their transcriptional profiles

**Figure 1:**
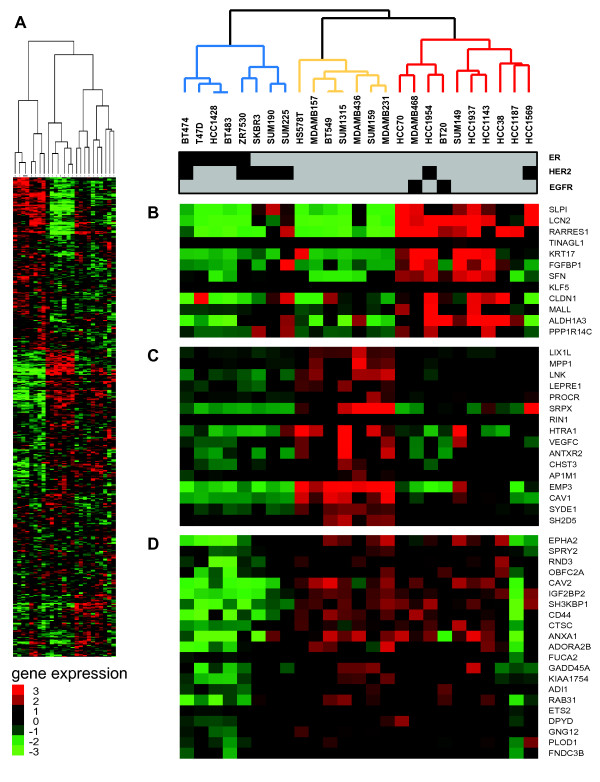
**Subtype-specific gene expression and molecular characteristics of breast cancer cell lines, using the Illumina HumanWG-6v2.0 microarray platform.** (**A**) “Two-way” hierarchical clustering of 25 BC cell lines and 5,693 variably expressed genes segregate into three groups, “Cluster 1, 2 and 3” indicated as blue, orange and red dendrogram branches. (**B**) Selected cluster commonalities in gene expression for ”Cluster 2”; (**C**) “Cluster 3”; and (**D**) both cell lines clusters are shown. Bar below dendrogram indicates phenotypic features of cell lines for ER, HER2 and EGFR.

To investigate the molecular heterogeneity of triple-negative BC cell lines and their representativeness of triple-negative breast cancers, we used Illumina HumanWG-6v2.0 to survey the phenotypic and genotypic characteristics of seven ER-negative mesenchymal BC cell lines (Hs578T, BT549, MDAMD157, MDAMD231, MDAMD436 and SUM159) and compared them with 13 ER-negative epithelial-like BC cell lines (BT20, HCC38, HCC70, HCC1143, HCC1937, MDAMD468, SUM149, SKBR3, SUM190, SUM225). Five ER-positive BC cell lines (T47D, BT474, ZR7530, BT483 and HCC1428) were also included in our dataset, to evaluate ER-responsive transcriptional signatures in ER-negative BC cell lines. In addition, two ER-negative/ HER2-positive epithelial BC cell lines were included (i.e. HCC1954 and HCC1569), and were employed as comparators with other triple-negative BC cell lines. Molecular pathological features of all BC cell lines are provided in Additional file 1 Table S1. Unsupervised hierarchical clustering of 5,693 highly variable Ensembl genes separated ER-negative BC cell lines into three groups (Figure 1). One group, designated “Cluster 1” (blue lines, Figure 1) included three ER-negative/HER2-positive BC cell lines, namely SKBR3, SUM190 and SUM225, which clustered with ER-positive/HER2-negative (T47D, HCC1428 and BT483) and ER-positive/HER2-positive (BT474, ZRF7530) cell lines. “Cluster 2” was uniformly composed of ER-negative/HER2-negative BC cell lines (orange lines, Figure 1) and was in complete concordance with the Basal “B” BC cell line subtype described in previous BC cell line studies [16-18]. “Cluster 3” consisted of cell lines (red lines, Figure 1) either having a triple-negative phenotype or showing amplification and higher abundance of HER2 (HCC1954, HCC1569) and EGFR gene amplification (BT20, MDAMB468). Investigating the expression levels of known triple-negative BC-related genes demonstrated subtle differences within these three groups. While “Cluster 3” cell lines expressed genes commonly found in the intrinsic gene list preferentially expressed in basal-like primary BC (*e.g. LCN, RARRES1*, *CLDN1*, *KRT17*; Figure 1B) [20], “Cluster 2” was characterised by a higher abundance of *CAV1*, a marker for the basal-like phenotype of sporadic and hereditary breast cancers [21], and the lymphangiogenic factor *VEGFC,* a potential therapeutic target for triple-negative BCs [22] (Figure 1C). Other genes previously associated with triple-negative BCs such as *c-MET*, *CD44* and *CAV2*[23,24] exhibited higher expression in both groups in comparison to “Cluster 1” cell lines (Figure 1D). 

### Representation of ER-negative breast tumour-related gene signatures in BC cell lines

**Figure 2:**
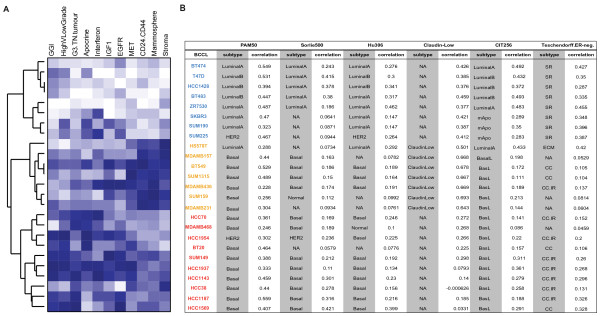
**Representation of gene signatures across BC cell lines.** BC cell lines are ranked based on their gene expression clustering. (**A**) Expression levels of eleven gene signatures previously associated with ER-negative BC. Similarities between gene signature and each BC cell line transcriptome was established as described in Material and Methods, and used for ranking lines. Each coloured square represents the rank (between 1 and 25) of the cell line to the specific gene signature, whereby blue indicates the highest rank (best resemblance to the gene signature), while white being the lowest rank. Gene signatures used were: G3.TN.Tumour [43]; Apocrine.Basal [9]; Interferon [28]; IGF1 [39]; EGFR [41]; c-MET [42]; Mammosphere [40]; CD24.CD44 [44]; HighGradeVLowGrade [47]; GGI [68]; Stroma [45]. (**B**) Classification of BC cell lines to BC subtypes by nearest centroid correlation based on gene expression signatures: PAM50 [26]; Sorlie500 [27]; Hu306 [69]; Claudin.Low [8], Teschendorff.ER.neg [25]; CIT256 [10]; both subtype assignment and Spearman correlation are presented.

Given that the combined expression pattern of certain genes can uniquely characterise different BC subtypes or can be used as surrogate markers for pathway activation, we selected a compendium of gene signatures representing various features of triple-negative BCs. Firstly, we investigated the representation of eleven gene signatures based on a “relative similarity score” and ranked the BC cell lines accordingly (Additional file 2 Table S2). Overall “Cluster 3” cell lines (red names, Figure 2) showed a good representation of gene signatures identified for “Apocrine”, G3.TN.Tumour” and high-grade breast carcinomas, while “Cluster 2” cell lines exhibited high similarities with the “Stroma”, “Mammosphere” and “CD24.CD44” expression patterns (orange names, Figure 2). The resemblance to the remaining four gene signatures showed a less striking concordance with the gene expression defined clusters, and identified SUM1315, HCC1569, SUM149 and HCC1143 cell lines as the best representatives for the “MET”, “EGFR”, “IGF1” and “Interferon” gene signatures, respectively. Secondly, we examined the 5 different ER-negative BC subgroups associated with a prognostic outcome [25] in the BC cell line transcriptomes based on centroid correlation classification. As shown in Figure 2B, “Cluster 3” cell lines had activation of the “cell cycle and cell proliferation pathway/immune response genes” and the “cell cycle and cell proliferation pathway” groups (Figure 2B), both of which showed association to basal-like BCs in their study [25]. In contrast, “Cluster 1” cell lines displayed highest correlation to ER-negative tumours of the steroid response group, supporting the outcome of our hierarchical clustering in which these cell lines group with the ER-positive cell lines (Figure 1). Lastly, to explore the representation of the molecular basal-like BC subtype within this BC cell line set, we performed nearest centroid classification using the class centroids from Parker *et al*. [26], Sorlie *et al*. [27], Hu *et al*. [28], Prat *et al*. [8] and Guedj *et al*., [10] (referred to as PAM50, Sorlie500, Hu306, Claudin.Low and CIT256 respectively, in Figure 2B). As expected given the manner in which the “Claudin.Low” gene signature was originally established [8], “Cluster 2” cell lines (Hs578T, MDAMB157, MDAMB231, MDAMB436, BT549, SUM159 and SUM1315) are representatives of the described “Claudin.Low” subtype. The recently published CIT classification [10] assigned all “Cluster 2” and “Cluster 3” cell lines to the basalL group (Figure 2B) with the exception of Hs578T which was assigned to the “LuminalA” subtype. Interestingly, in “Cluster 1”, ER-negative/HER2-positive cell lines SKBR3, SUM190 and SUM225 were labelled as mApo (‘molecular Apocrine’) groups, in agreement with ER-negative/HER2-positive breast carcinomas in this molecular subtype [9]. PAM50 classification assigned all “Cluster 2 and 3” BC cell lines except Hs578T and HCC1954 to the basal-like BC subtype. Assignments of cell lines into the basal-like subtype with class centroids obtained from Sorlie500 and Hu306 were in good agreement with those obtained with PAM50, however only 2 out 10 cell lines were consistently classified as of luminal A, luminal B or HER2 by all methods. To determine the agreement of these three centroid classifications, we used the free-marginal Kappa statistics of Brennan and Prediger [29] and saw substantial agreement in the classification of the basal-like (Kappa score = 0.78), luminal A (Kappa score = 0.7) and HER2 subtypes (Kappa=0.63), in agreement with the results of previous studies [7,30]. 

### Copy number aberrations and associated gene expression changes in BC cell lines represent those observed in triple-negative BCs

**Figure 3:**
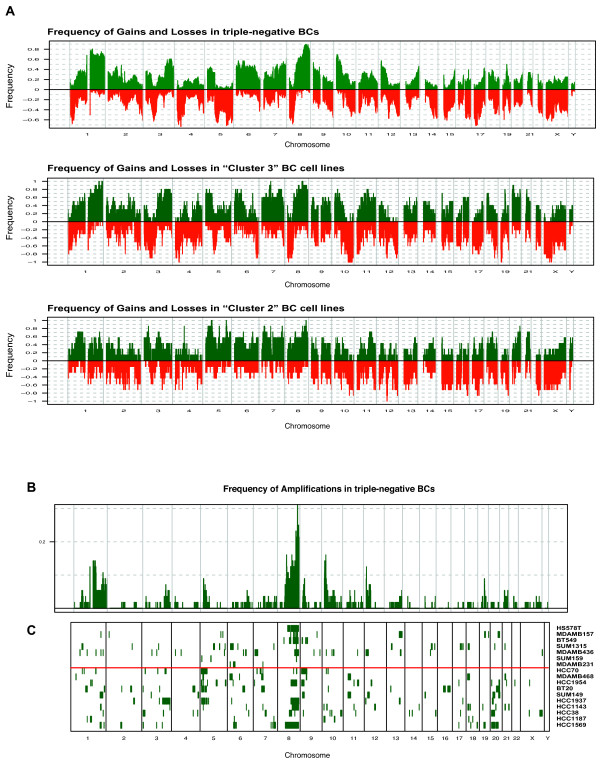
**Genomic alteration of triple-negative BCs and ER-negative BC cell lines.** (**A**) Genomic profiles of 56 triple-negative BCs were obtained from Turner [31]. The proportion of samples for each group (triple-negative BCs, ”Cluster 2 and 3” BC cell lines) is plotted in which each genomic area is gained (green) or lost (red) according to their genomic position. (**B**) The frequency of triple-negative BCs in which the smoothed log2ratios of each BAC clone above 0.45 is plotted (y-axis) according to its genomic location (x-axis). (**C**) Using the same criteria for the aCGH data of BC cell lines, amplified BAC clones for each BC cell line (row) are represented as green lines along the genome. The separation between ”Cluster 2 and 3” BC cell lines are indicated with a red line.

**Figure 4:**
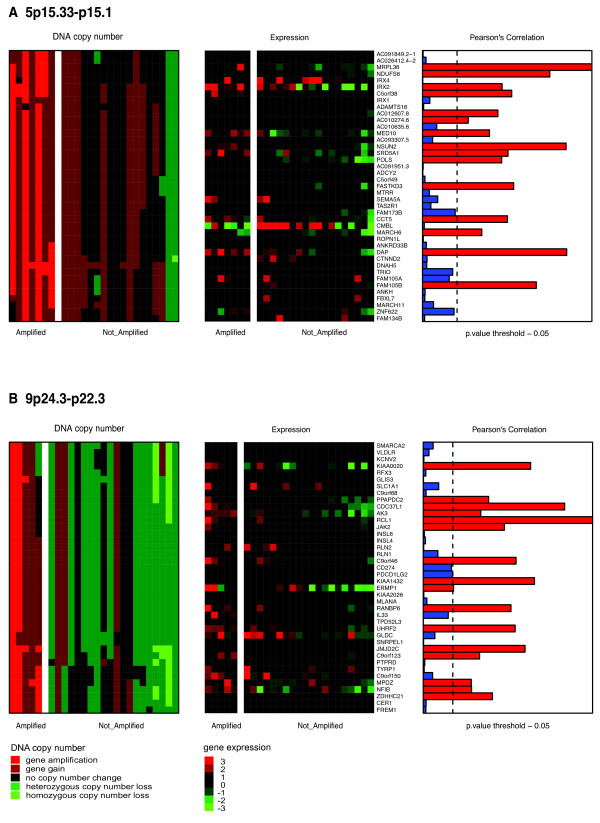
**BC cell lines recapitulate recurrent triple-negative BC-specific amplicons and their possible drivers.** Matched heatmaps of gene expression and aCGH within regions of recurrent amplification in BC cell lines (**A**) and (**B**). Cell lines were split into those with amplification (AMP) and those without (NA). Heatmap of aCGH (left) shows amplification in red, deletion in green and no change in black for each corresponding chromosomal location of the respective gene. Expression heatmap (middle) indicates if expression values for the gene are above (red) or below (green) the median value. Barplots (right) illustrate of a Pearson’s correlation analysis between expression and DNA copy number levels. Black dotted lines show adjusted *P_value* threshold of 0.05.

To identify BC cell lines harbouring copy number aberrations specific for triple-negative BC, we performed aCGH using a 32k tiling path array platform and surveyed their genomic changes (individual aCGH-profiles are provided as Additional file 3 Figure S1). By grouping the BC cell lines based on the three expression clusters, “Cluster 2” cell lines displayed significantly less high-level amplifications and deletions compared with the other two clusters (Additional file 4 Figure S2). Next, we retrieved CNAs identified in our previous study on 56 triple-negative BCs [31], analysed on the same genomic platform. Overall the frequency of gains and losses seen in triple-negative BCs was more similar to “Cluster 3” than to “Cluster 2” and “Cluster 1” cell lines (Figure 3A). Recurrent amplification seen in primary triple-negative BCs were recapitulated in at least one BC cell line (Additional file 5 Table S3). The most highly recurrent triple-negative BC-specific amplicons in BC cell lines were on 5p15.33-p15.1 (HCC1143, HCC1937, HCC1954, HCC70, MDAMB468 and BT20), followed by 9p24.3-22.3 and 7q11.1 found in 6 “Cluster 3” cell lines and 4 “Cluster 3” cell lines, respectively (Figure 3B). Given that these regions are also characterised by common polymorphisms, genes such as *JAK2* (9p24) [32], *NUNS2* (5p15) [33] or *LIMK1* (7q11) [34] previously associated with breast cancers, and gained preferentially in basal-like breast cancers [35] might validate that these regions contain genes providing a selective advantage for triple-negative BCs. A comprehensive integration of genes lost or gained in the 2,000 breast cancer study is provided in Additional file 6 Table S4. Amplifications on 3q24-q25.1, 3q25.32-q25.33, 5p14.3-p14.1, 7p11.2 and 9p22.3 were observed only in ”Cluster 3” cell lines, and the 13q32.3-q33.3 amplicon only in “Cluster 2” cell lines, illustrating a good representation of triple-negative BC-specific CNAs in ER-negative cell lines, with some of them having a higher prevalence in one than the other expression defined group. We and others have previously demonstrated that the expression levels of certain genes located in triple-negative BCs specific CNAs is copy number dependent [31,36]. To investigate if these dependencies are also recapitulated in BC cell lines, we integrated expression data with cbs-smoothed aCGH profiles of each BC cell lines. Using Pearson’s correlation (fdr adjusted *P_value* <0.05), 4,571 genes showed significantly correlation between their expression and DNA copy number levels (Additional file 6 Table S4). This set encompassed 1,158/2,064 triple-negative BC copy-number dependent genes as determined by Turner [31], and included genes such as transcriptional regulators (n=98), kinases (n=47), phosphatases (n=24) and transmembrane receptors (n=5), as well as biomarkers for diagnosis (n=42), prognosis (n=14), disease progression (n=7) and known drug targets (n=20) (Additional file 5 Table S4). Triple-negative BC–specific amplicons, recurrently amplified in our BC cell lines (*e.g.* 5p15.33-p15.1 and 9p24.3-22.3) and harbouring genes with DNA copy number-dependent expression levels are shown in Figure 4. Among those were genes, such as *JAK2*, *NSUN2* and *NFIB* previously shown to have pathogenic roles, but also novel potential drivers e.g. *PPAPDC* or *RANBP6*, which could be selectively required for the survival of cells harbouring those amplification (Figure 4). 

### Epigenetic influence on sub-type specific genes in ER-negative BC cell lines

**Figure 5:**
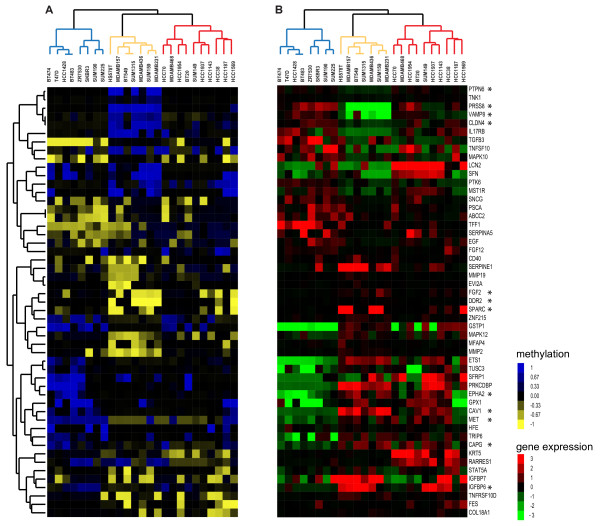
**Matched heatmaps of gene expression and methylation profiling.** 51 genes whose epigenetic-regulated expression varied between the three expression clusters. (**A**) Methylation heatmap (left) illustrates unmethylated probes in yellow, methylated in blue and partially methylated in black. (**B**) Expression heatmap displays the mean centred expression level for the corresponding gene. BC cell lines are ordered based on the gene expression clustering, and groups are illustrated as blue, orange and red dendrogram branches for “Cluster 1, 2 and 3” BC cell lines, respectively. Genes of the Claudin-low signature are indicated with asterisks.

The functional validity of the methylation pattern found on CpG islands in cultured cancer cell lines has been the matter of controversy [37]. To study epigenetic patterns in BC cell lines, we produced genome-wide methylation profiles on Illumina GoldenGate bead arrays. Unsupervised hierarchical clustering using 1,223 CpG probes, corresponding to 707 genes, revealed a different grouping (Additional file 7 Figure S3) as it was observed when their expression profiles were clustered (Figure 1). To validate that the BC cell line specific methylation pattern can be also found in triple-negative BC, we retrieved methylation data of 189 fresh-frozen BCs performed on the same microarray [38]. Due to the lack of HER2 status information in their study, 43 basal-like BCs were used as surrogates for triple-negative tumours and 165 CpG islands with variable methylation levels were observed. Of those 165, 128 CpG probes showed also changes in their methylation status in our BC cell lines (Additional file 6 Table S4). Investigating the methylation state of these CpG islands in each BC cell line individually, demonstrated an overall good representation of ≥70% of hypo- and hyper- basal-like BC-specific methylation events specifically in ER-negative BC cell lines (Additional file 8 Figure S4). Given that BC subtype specific expression has been suggested to be under epigenetic influence [38], we surveyed the methylation effect on gene expression in BC cell lines. Integration of methylation and gene expression data resulted in 1,129 CpG gene pairs (corresponding to 652 genes) and identified an inverse correlation between methylation and gene expression levels for 93 pairs (correlation < 0.55; adjusted *P_value* 0.05). Performing bootstrap analysis by randomly sampling the BC cell lines, we showed that the number of significant association between gene expression and methylation was more than 90 fold higher than expected. Using these 93 CpG gene pairs in a multiclass SAM analysis revealed 73 with specific methylation patterns over the three expression clusters, particularly distinguishing “Cluster 2” cell lines from the others (Figure 5). As described previously, “Cluster 2” cell lines exhibited among others, expression patterns similar to the “Claudin.low” gene signature [8]. Fifteen genes of the “Claudin.low” gene signature had CpG sites with varying methylation pattern over these BC cell lines which was significantly higher than expected by chance (hypergeometric testing *P_values* < 0.001), whereby genes downregulated in “Claudin.low” cancers according to “Claudin.low” signature were methylated and vice-versa, such as “Claudin.low” signature genes such as *PRSS8*, *CLDN4* and *VAMP8* were downregulated in “Cluster 2” and their CpG islands were methylated, while genes like *SPARC* or *DDR2* had unmethylated CpG islands and showed higher abundance in these BC cell lines. By comparing those with epigenetic-regulated genes in breast cancers [38], we identified 12 genes with the same concordant pattern (asterisks, Figure 5). Taken together, our analyses demonstrate that BC cell lines retain methylation–dependent gene expression patterns observed in basal-like BCs, and strengthen an epigenetic influence on some BC phenotypes that are retained in their equivalent model systems. 

## Discussion

Triple-negative BCs represent a heterogeneous group with diverse deregulation of biological pathways. Here, we extended the molecular characterisation of ER-negative BC cell lines based on their genetic, epigenetic and transcriptional profiles, and correlated these with a comprehensive compendium of gene signatures reflecting different features of ER-negative BCs [8,9,28,39-45]. Initial cluster analysis of BC cell lines’ expression profiles resulted in three groups, two clusters encompassing purely ER-negative BC cell lines (“Cluster 3” and “Cluster 2”), while one consisted of three ER-negative and all ER-positive BC cell lines. The first two cell line clusters were in good agreement with recent BC cell line studies [6,11,15-18,31,46]. While “Cluster 2” encompassed cell lines that were all represented in the Basal “B” cluster of Neve *et al.*[18] and were assigned to the triple-negative mesenchymal phenotype by Lehmann *et al*. [11], most of the “Cluster 3” cell lines were part of Neve’s Basal “A” cluster [18] and part of the basal-like subtype according to Lehmann *et al*., [11]. Our “Cluster 3” cell lines exhibited expression patterns found in transcriptional profiles of microdissected grade 3 triple-negative breast tumours [43] as well as grade 3 versus grade 1 breast carcinomas [9,47]. HCC1143, an ER-negative/HER2-negative cell line, was the top *in vitro* representative “Cluster 3” cell line for the triple-negative phenotype of microdissected grade 3 triple-negative breast tumours [43]. The transcriptional profile of HCC1143 also seemed very suitable in modelling the Interferon, *IGF1* and *MET* signalling pathways. BC cell lines with expression patterns most closely associated with the Apocrine.Basal subtype [9] were not defined to one or the other cluster and HCC1954, an ER-negative/HER2-positive cell line of “Cluster 3” displayed the highest representation. These BCs were originally defined on the basis of their androgen receptor level and many of them harboured ERBB2 amplifications [9]. This is in agreement with our findings, whereby using a recently published BC classifier, named CIT, three ER-negative/HER2-positive cell lines SKBR3, SUM190 and SUM225 were classified to the mApo (molecular Apocrine) breast cancer subtype [10]. In a study, MDAMB453, SUM185, CAL148 and MFM223 showed expression patterns associated with androgen receptor signalling and were more sensitive to androgen receptor antagonist bicalutamide and an Hsp90 inhibitor [11]. While none of those cell lines were part of our study, BT549 and HCC1937, BC cell lines used in our study and good representatives of the Apocrine.Basal subtype showed high sensitivity to Hsp90 inhibitors in Lehmann’s work [11]. The Claudin-low subtype has been described as BC entity [8,48], which is enriched for ER-negative invasive ductal carcinomas, while displaying low levels of luminal differentiation markers and activation of pathways involved in epithelial-to-mesenchymal transition, stem cell-like features and the immune response [8]. Integration of gene expression with methylation data over BC cell lines revealed a group of CpG islands corresponding to genes within the Claudin-low signature, showing an inverse correlation between their methylation and the genes expression in BC cell lines and BCs [38]. Our findings are in agreement with those from a recent report that led to the identification of a set of genes whose expression was epigenetically regulated and when used as a gene signature identified mesenchymal features in Claudin-Low breast tumours [19]. Furthermore, they postulated that a deviant methylation might reflect cell lineage commitment in agreement with our hypothesis of a contribution of an epigenetic regulation to the Claudin-Low subtype. Aberrant DNA methylation events have initially been thought to accumulate in a random fashion within cells in pre-malignant tissues, however, lately it has also been shown that *de novo* methylation has a predictable pattern, creating plasticity followed by commitment to alternative cell lineages [49]. Holm and colleagues proposed that BC subtypes might be driven by different epigenetic events and could reflect their different cellular origins [38]. Nevertheless, an alternative hypothesis might also be that the methylation patterns are a result from mutations in genes controlling the epigenetic landscape in breast cancer [50]; thus further investigation is warranted to determine whether these distinctive methylation patterns are results of genetic aberration in epigenetic regulator genes and/or contribute to delineation of the differentiation hierarchy of Claudin-Low and other BC subtypes.

We and others have recently shown that basal-like BCs are most likely derived from luminal progenitor cells [51,52]. Identifying *in vitro* models would enhance our understanding of these cell populations. Interestingly, our cluster and gene signature analysis revealed ER-responsive features for SKBR3, SUM190 and SUM225, three ER-negative/HER2-positive cell lines. SKBR3 cells are well known to have luminal BC characteristics [53]. In contrast, the classification of SUM190 and SUM225 is controversial. While some BC cell line studies assigned them to basal-like cell lines [15,18], others supported our finding of SUM190 within the ER-positive cluster [16]. SUM225, although not included in this study, was classified as of luminal phenotype in other studies [54]. Common to both is the expression of luminal cytokeratins 8, 18 and 19 [55] as well as genes found in luminal progenitor cell population (data not shown) [51], more consistent with a luminal classification. Although SUM225 was found to highly express ALDH1, a marker for the so-called BC stem cells [56], further investigations are necessary to ascertain whether SUM190 and SUM225 represent appropriate *in vitro* models for luminal intermediate progenitor populations.

High-level amplifications are less likely to represent random aberrations and often encompass genes driving the development or maintenance of tumour growth. Three-quarters of triple-negative BCs harbour at least one amplicon [31], however, their recurrence rates are lower than those of high-level CNAs found in ER-positive/ HER2-negative and HER2-positive BC subtypes (*e.g. ERBB2*-amplicon in HER2, and *CCND1* and *FGFR1* in luminal breast tumours [57]). Here, we demonstrated that triple-negative BC-specific amplicons are recapitulated in ER-negative BC cell lines and that some of them are associated with higher frequencies either to ”Cluster 2” or “Cluster 3” expression clusters. For example, the region on 5p15.33-p15.1 was found to be recurrently amplified in 5/56 and 10/28 triple-negative BCs [31,36], was present in six ”Cluster 3” but only in one ”Cluster 2” cell lines. Notably, these genomic sites map to regions of common germline copy number polymorphism and the functional consequences of their increased DNA levels require further validation. Nevertheless, several genes located within these amplified regions were found gained with a higher frequency in basal-like BCs in a recent study investigating 2,000 breast tumours [35] and expression levels significantly correlated with their DNA copy number in triple-negative BC cell lines and tumours for several of these genes [31]. A recent study investigated genes on 5p15.33-p15.1 in more detail and showed that silencing of the overexpressed and amplified *NUNS2*, a *MYC* target gene, reduced cell number in some BC cell lines [33]. *NUNS*2 expression has been found significantly increased in malignant tissues whereas it could only be found in testis in normal tissues, furthermore its role in stabilising the mitotic spindle and phosphorylation by Aurora-B make it an interesting target for cancer diagnostics and molecular therapeutics.

## Conclusion

Taken together, transcriptional, genomic and epigenetic profiles of 25 BC cell lines, enriched for those representing triple-negative features, help to define cell lines that most closely capture individual examples of the heterogeneous characteristics within triple-negative BCs. By cross-referencing different high-resolution datasets, we provide useful resources to further study transcriptional, as well as genetic and epigenetic modulation and inform the best selection of available *in vitro* models for the identification and validation of potential novel therapeutic targets relevant to triple-negative BCs.

## Methods

### BC cell lines

BT20, BT474, BT483, BT549, Hs578T, MDAMB157, MDAMB231, MDAMB436, MDAMB468, T47D, SKBR3, ZR75-30, HCC1937, HCC70, HCC1428, HCC1143, HCC38, HCC1187, HCC1569, HCC1954 were obtained from ATCC (Manassas, VA, USA). SUM159, SUM149, SUM1315, SUM225, SUM190 were purchased from Asterand plc (Detroit, MI, USA) (Additional file 1 Table S1). All lines were grown according to the supplier’s recommendation and authenticated by means of Short Tandem Repeat (STR) analysis (PowerPlex® 1.2 System, Promega, WI, US) as previously described [58]. STR profiles were matched to the German Collection of Microorganisms and Cell Cultures (DSMZ)–database (http://www.dsmz.com). BC cell lines were stratified into mesenchymal and epithelial-like morphological groups based on previous studies [11,16,18].

### RNA and DNA isolation

Cells were grown to ~70% confluence before harvesting nucleic acids. DNA was prepared using the Qiagen DNeasy tissue kit (Qiagen, Valencia, CA) and RNA was isolated using Trizol (Invitrogen, Carlsbad, CA) according to the manufacturers’ protocol. DNA concentration was measured with Picogreen (Invitrogen, Paisley, UK). Integrity of RNA was quantified using the Agilent 2100 Bioanalyser with RNA Nano LabChip Kits (Agilent Biosystems, Foster City, CA).

### Microarray analyses

Analyses of microarray data were performed in the R environment 2.12.0 (http://www.r-project.org/) making use of several Bioconductor packages (http://www.bioconductor.org/). All Microarray probes and external gene signatures were mapped to the Ensembl 55 (human genome build 37) to ensure uniform annotation. Microarray data have been deposited in Array Express (E-TABM-928; http://www.ebi.ac.uk/arrayexpress/). A Sweave document describing the statistical analysis is provided as Supplemental Methods (Addition file 9).

### Gene expression profiling

Using the Illumina Totalprep RNA amplification kit (Ambion, UK), 200ng total BC cell line RNA was amplified and hybridised to Illumina HumanWG-6v2.0 arrays gene expression bead-chips at Genizon BioSiences Inc (Quebec, CA). Raw data obtained from Illumina BeadStudio (Illumina, San Diego, CA) were preprocessed using the “lumi” -Bioconductor package [59]. Microarray probes absent in more than 80% of samples based on an Illumina BeadStudio detection *P_value* >0.01 were removed from further analysis. For unsupervised hierarchical clustering of gene expression, 5,693 unique Ensembl genes with a median absolute deviation (MAD) of ≥0.4 across all BC cell lines were selected. Ward clustering was applied to genes and arrays after median centring using Pearson’s correlation as a distance measurement and 10,000 bootstrap iterations were performed to assess the significance of the observed the stability of the clusters using the pvclust package for R [60]. Resulting clusters were visualised with Java TreeView [61]. Two strategies were applied for gene expression signature analysis: (1) When centroids for specific classes (*e.g.* BC subtypes or groups of ER-negative breast tumours [25]) were publicly available, assignment of BC cell lines to these classes was based on their highest Spearman rank correlation. Classification included class centroids defined by Sorli*e*[27], Hu [28], Parker [26], Prat [8], CIT256 [10] and Teschendorff [25]. (2) To monitor specific ER-related features, 11 gene signatures were retrieved from publication (see Additional file 3 Table S2 for a detailed description). For the “G3.TN.Tumour” signature, we used our previously published expression data of microdissected breast tumours [43]. Significance Analysis of Microarrays (SAM) [62] with 1,000 permutations and 0% fdr was used to identify significant genes for triple-negative BCs, using a two-class comparisons between tumours belonging to the triple-negative subtype and all other subtypes. For each BC cell line, a weighted mean expression of genes present in the respective signature was determined, and cell lines were ranked based on their concordance.

### Array-based comparative genomic hybridisation (aCGH)

Labelling, hybridisation, image and initial data analysis of the 32k BAC tiling path aCGH platform, produced at the Breakthrough Breast Cancer Research Centre, London, UK [63] was carried out as previously described [43]. Breakpoint analysis was performed using the circular binary segmentation (cbs) algorithm [64] and rescaled such that the genome MAD was the same in each sample. Only segments of ≥ 3 BAC clones were used in further analyses. Thresholds for cbs-smoothed data were estimated as described previously [65]. Briefly, cbs-smoothed aCGH Log_2_ values <−0.08 were classified as losses, >0.08 but ≤0.45 were categorised as gains, and >0.45 were referred to as high-level gains/ amplifications. To determine genomic instability, the fraction of amplified, deleted or total BACs over the whole data set was calculated and presented as a proportion. Gene expression values were compared with median cbs-smoothed aCGH data for all BACs encompassing the genomic position using Pearson’s correlation adjusted for multiple testing [66]. Matched heatmaps between gene expression and genomic data were created as described in [31] showing the minus log_10_ Pearson’s *P_value* of each gene-aCGH pair correlation. The raw and cbs-smoothed aCGH data are deposited at http://rock.icr.ac.uk/collaborations/GrigoriadisA/.

### Methylation array analysis

Hybridisation and image analysis of the Illumina GoldenGate methylation beadarrays were performed at the Genome Centre (Barts and the London School of Medicine and Dentistry, London, UK). Methylation profiles of the BC cell lines, obtained through the BeadStudio Methylation Module (Illumina, San Diego, CA), was normalised by dichotomising the un- /methylated CpG islands separately before equalising their median according to the “methylumi” package (http://www.bioconductor.org/). CpG sites located on the X chromosomes were removed, as well as constitutively un-/ methylated probes, resulting in 1,223 CpG sites (data are available at http://rock.icr.ac.uk/collaborations/GrigoriadisA/). The methylation state of CpG islands given as a ß-value [67] was stratified into three categories: ß-values ≤ 0.25, ≥ 0.75 and between ≥ 0.25 and ≤ 0.75; and interpreted as un-/, methylated and partially methylated CpG sites, respectively. These cut-offs are slightly more stringent than Holm *et al.* has used them for the analysis of breast carcinomas using the same methylation array platform [38] to increase the chances of true-positive events. Initial analysis revealed a similar methylation frequency in all BC cell lines, determined as the fraction of methylated CpG sites, affecting on average 31% of all CpG islands. Using a total of 10,000 permutations to obtain reasonable estimates of dependencies, sample labels were permuted and correlation analyses between gene expression and methylation values were carried out on the resampled data set.

## Abbreviations

BC: Breast cancer; ER: Estrogen-receptor; PR: Progesterone receptor; CNAs: Copy Number Aberrations; MAD: Median Absolute Deviation; SAM: Significance Analysis of Microarrays; ACGH: Array-based comparative genomic hybridisation; CBS: Circular binary segmentation; BAC: Bacterial Artificial Chromosome; AMP: Amplification.

## Competing interests

The authors have declared no conflict of interests.

## Authors' contributions

Conceived and designed the experiments: AG, AM, EN, RN, JSR-F and AT. Performed the experiments: PJW, RN and JF. Analysed the data: AG, AM. Wrote the paper: AG, EN, JSR-F and AT. All authors read and approved the final manuscript.

## Supplementary Material

Additional file 1 **Table S1.** Clinicopathological features of breast cell lines. Clinicopathological characteristics of BC cell lines. Click here for file

Additional file 2 **Table S2.** Gene signatures with relevance in ER-negative breast tumours. Compendium of gene signatures, listing their genes, their citation and their relevance for triple-negative BCs. Click here for file

Additional file 3 **Figure S1.** aCGH profiles Of BCCLs.zip. Folder provided aCGH-profiles for each BCCL individually. Gains are coloured in green, while copy number loss is shown in red. Click here for file

Additional file 4 **Figure S2.** Distribution of CNAs over 3 gene expression clusters. Genomic instability varies between different BC cell lines expression clusters. For each BC cell line the genomic instability was determined, defined as the fraction of altered genome, and compared between the three expression clusters. Total genomic aberrations, amplifications and deletions were investigated separately*. P_values* (Welch t-test) for pairwise comparison are shown in red. Click here for file

Additional file 5 **Table S3.** Recurrent amplicons of 56 Grade 3 TNBC in BCCLs. Recurrent amplicons of TNBC found in BC cell lines of “Cluster 1, 2 and 3”. Click here for file

Additional file 6 **Table S4.** Gene centric analysis in 25 BCCL. Gene centric table of BC cell lines, showing the copy number state of each gene in each BC cell line, their un/adjusted Pearson’s correlation between gene expression and copy number; their correlation between gene expression and copy number in triple-negative BCs (both taken from Turner [31] their methylation states in BC cell lines, their Pearson’s correlation between methylated state and their gene expression in BC cell lines and basal-like BCs [38]. Click here for file

Additional file 7 **Figure S3.** Hierarchical clustering of BCCL methylation data. Unsupervised hierarchical clustering of BC cell lines based CpG islands. BC cell lines of ”Cluster 1, 2, 3” are shown in blue, orange and red, respectively. Click here for file

Additional file 8 **Figure S4.** Distribution of methylated and unmethylated CpG islands in each BCCL. representation of BC specific un/methylated CpG sites in BC cell lines. Methylation marks for triple-negative BC were retrieved from Holm’s methylation profiling analysis [38]. Barplots represent the number of un/methylated CpG islands in each BC cell lines as identified of being un/methylated in BCs. BC cell lines of ”Cluster 1, 2, 3” are shown in blue, orange and red, respectively. The order of the BC cell lines is based on their gene expression clustering. Click here for file

Additional file 9 **Sweave Documentation.** Sweave documentation of analysis. Click here for file
