# Supplementary material for: Molecular characterisation of cell line models for triple-negative breast cancers
Source: BMC Genomics. 2012 Nov 14;13:619. doi: 10.1186/1471-2164-13-619 (PMC3546428; doi:10.1186/1471-2164-13-619)
Supplement: Additional file 1 — Table S1. Clinicopathological features of breast cell lines. Clinicopathological characteristics of BC cell lines. [file 1471-2164-13-619-S1.pdf]

Supplemental Table 1. Clinicopathological characteristics of breast cancer cell lines

Cell line	subtypeNeve*	ER*	PR*	HER2**	p53IHC*	p53*	EGFR[%]	ALDH1 activity[#]	PIK3CA[$]	CDKN2A[$]	PTEN[$]	Source[$$]	BRCA1[&]
BT20	basalA	-	-	N	+	wt	amp	positive	mut	mut	wt	PT	wt
BT474	luminal	+	+	amp	+		N		mut	wt	wt	PT	wt
BT483	luminal	+	+	N	-		N		wt	wt	wt	PT	wt
BT549	basalB	-	-	N	+	mut	N		wt	wt	mut	PT	wt
HCC1143	basalA	-	-	N	+	mut	N		wt	wt	wt	PT	
HCC1187	basalA	-	-	N	+	mut	N		wt	wt	wt	PT	
HCC1428	luminal	+	+	N	+		N		wt	wt	wt	PE	
HCC1569	basalA	-	-	amp	-	mut	N		wt	wt	**mut**	PT	
HCC1937	basalA	-	-	N			N	positive	wt	wt	mut	PT	mut
HCC1954	basalA	-	-	amp			N	positive	mut	wt	mut	PT	
HCC38	basalB	-	-	N	+	mut	N	positive	wt	mut	wt	PT	
HCC70	basalA	-	-	N	+	mut	N		wt	wt	mut	PT	
HS578T	basalB	-	-	N	+	mut	N	positive	wt	wt	wt	PT	wt
MDAMB157	basalB	-	-	N	-		N	positive	wt	wt	wt	MED.C	wt
MDAMB231	basalB	-	-	N	+	mut	N	positive	wt	mut	wt	MET.AC	wt
MDAMB436	basalB	-	-	N			N	positive	wt	wt	wt	MET.AC	mut
MDAMB468	basalA	-	-	N			amp		wt	wt	mut	MET.AC	wt
SKBR3	luminal	-	-	amp	+		N	positive	wt	wt	wt	PE	wt
SUM1315	basalB	-	-	N			N		wt	wt	wt		mut
SUM149	basalB	-	-	N			N	positive				PE	mut
SUM159	basalB	-	-	N			N	positive	mut	wt	wt		wt
SUM190	basalA	-	-	amp			N	negative	mut	wt	wt	PT	wt
SUM225	basalA	-	-	amp	+		N		wt	wt	wt		wt
T47D	luminal	+	+	N	+	mut	N	negative	mut	wt	wt	PE	wt
ZR7530	luminal	+	-	amp	-	wt	N	negative	wt	wt	wt	AF	wt

*Neve *et al*., 2006 // ** 32k aCGH BAC data // [$] http://www.sanger.ac.uk/genetics/CGP/cosmic // [#] Charafe-Jauffret *et al*., 2010
[%] Mackay *et al*., 2009 // [&] Elstrodt *et al*., 2006 // [$$] Kao *et al*., 2009
