# Supplementary material for: Molecular characterisation of cell line models for triple-negative breast cancers
Source: BMC Genomics. 2012 Nov 14;13:619. doi: 10.1186/1471-2164-13-619 (PMC3546428; doi:10.1186/1471-2164-13-619)
Supplement: Additional file 3 — Figure S1. aCGH profiles Of BCCLs.zip. Folder provided aCGH-profiles for each BCCL individually. Gains are coloured in green, while copy number loss is shown in red. [file 1471-2164-13-619-S3.pdf]

Add 6
