# Supplementary figures and images for: Molecular characterisation of cell line models for triple-negative breast cancers
Source: BMC Genomics. 2012 Nov 14;13:619. doi: 10.1186/1471-2164-13-619 (PMC3546428; doi:10.1186/1471-2164-13-619)

## Distribution Of CNAs Over 3 Gene Expression Clusters.

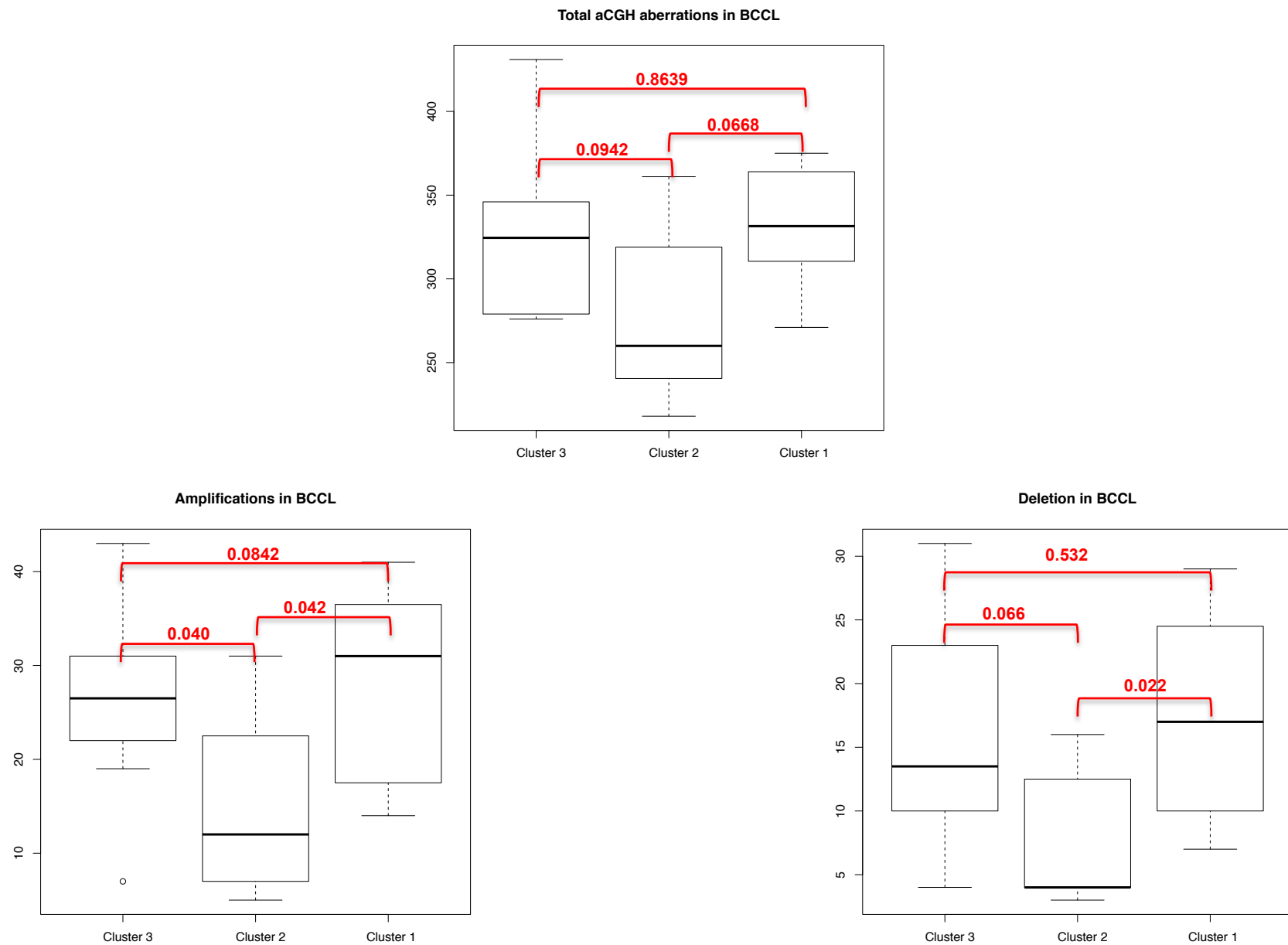

Supplement: Additional file 4 — Figure S2. Distribution of CNAs over 3 gene expression clusters. Genomic instability varies between different BC cell lines expression clusters. For each BC cell line the genomic instability was determined, defined as the fraction of altered genome, and compared between the three expression clusters. Total genomic aberrations, amplifications and deletions were investigated separately. P_values (Welch t-test) for pairwise comparison are shown in red. [file 1471-2164-13-619-S4.pdf]

# Hierarchical Clustering of BCCL Methylation Data

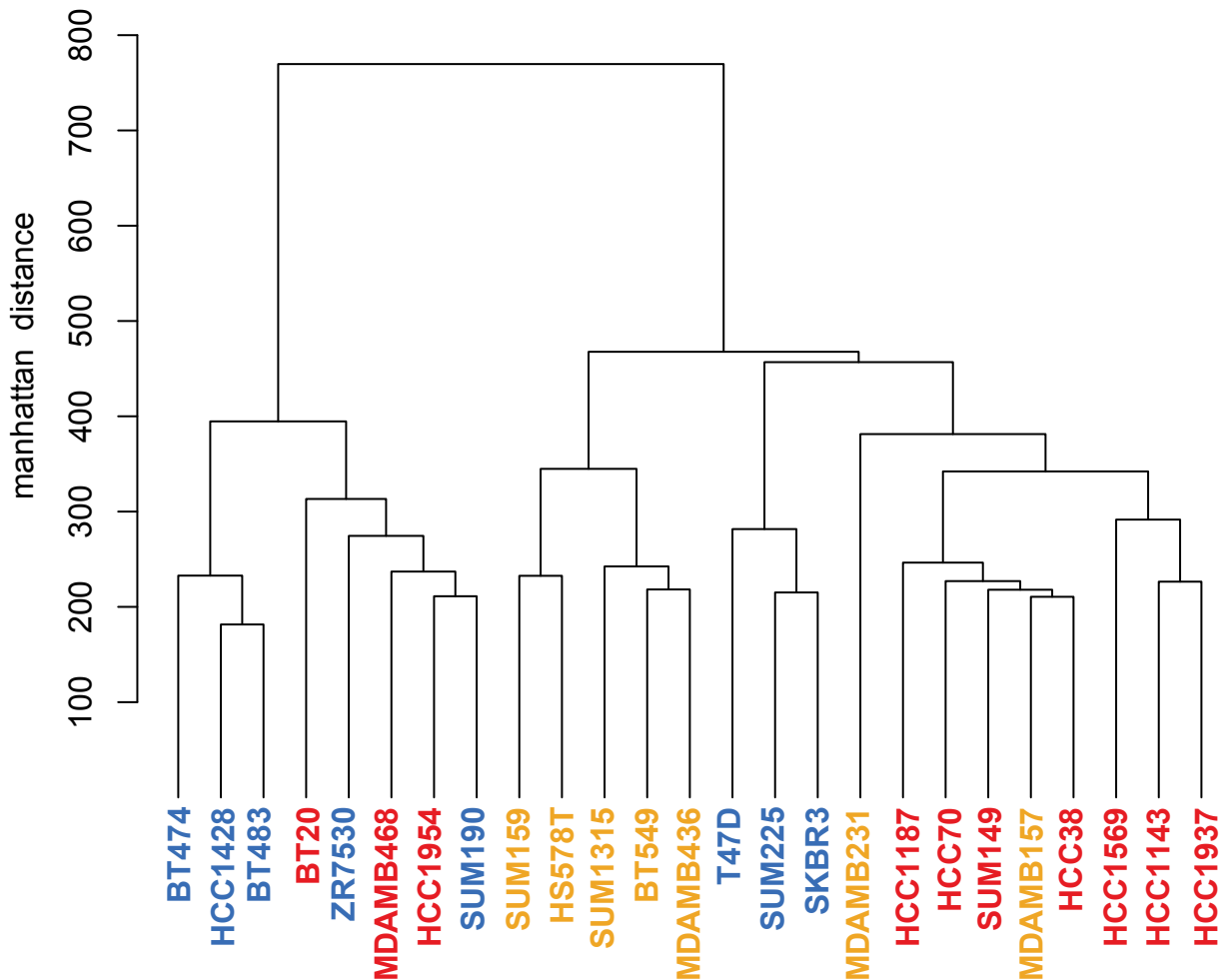

Supplement: Additional file 7 — Figure S3. Hierarchical clustering of BCCL methylation data. Unsupervised hierarchical clustering of BC cell lines based CpG islands. BC cell lines of ”Cluster 1, 2, 3” are shown in blue, orange and red, respectively. [file 1471-2164-13-619-S7.pdf]
