# Supplementary material for: Molecular characterisation of cell line models for triple-negative breast cancers
Source: BMC Genomics. 2012 Nov 14;13:619. doi: 10.1186/1471-2164-13-619 (PMC3546428; doi:10.1186/1471-2164-13-619)
Supplement: Additional file 8 — Figure S4. Distribution of methylated and unmethylated CpG islands in each BCCL. representation of BC specific un/methylated CpG sites in BC cell lines. Methylation marks for triple-negative BC were retrieved from Holm’s methylation profiling analysis [38]. Barplots represent the number of un/methylated CpG islands in each BC cell lines as identified of being un/methylated in BCs. BC cell lines of ”Cluster 1, 2, 3” are shown in blue, orange and red, respectively. The order of the BC cell lines is based on their gene expression clustering. [file 1471-2164-13-619-S8.pdf]

Distribution of Methylated and Unmethylated CpG islands in BCCLs

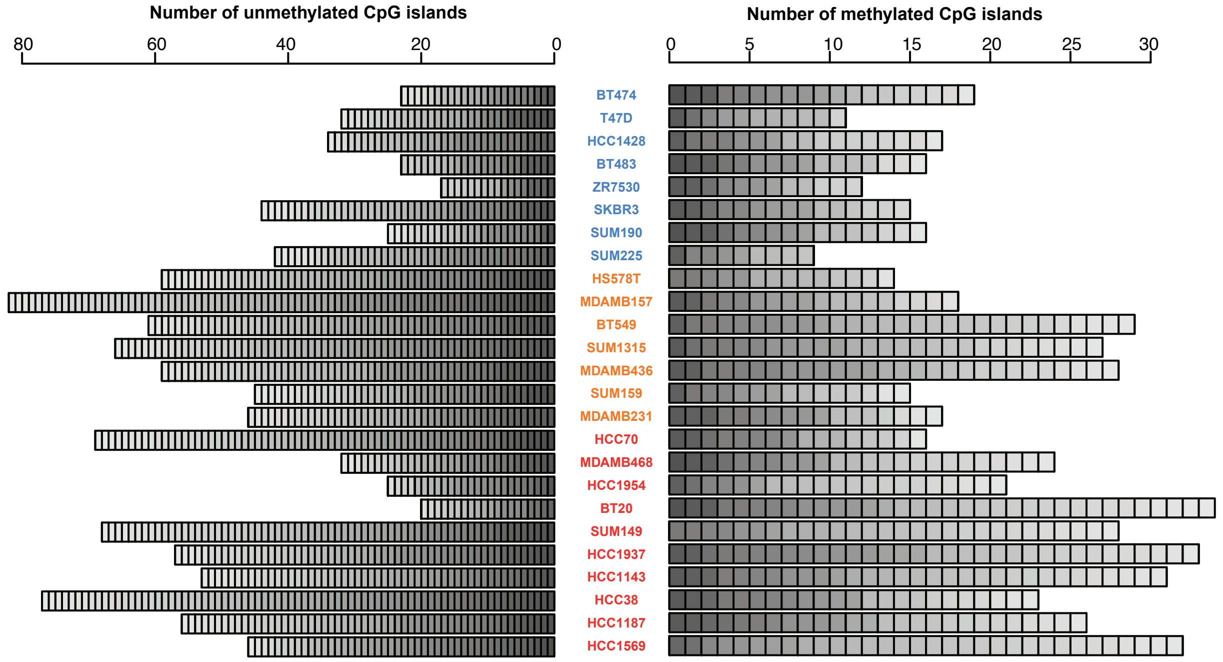

Grigoriadis et al., Supplemental Figure 4
