# Supplementary material for: Molecular characterisation of cell line models for triple-negative breast cancers
Source: BMC Genomics. 2012 Nov 14;13:619. doi: 10.1186/1471-2164-13-619 (PMC3546428; doi:10.1186/1471-2164-13-619)
Supplement: Additional file 9 — Sweave Documentation. Sweave documentation of analysis. [file 1471-2164-13-619-S9.pdf]

Molecular characterisation of cell line models for triple negative breast cancers

Anita Grigoriadis and Alan Mackay

2012-05-20

Triple-negative breast cancers (BC) represent a heterogeneous subtype of BCs, generally associated with an aggressive clinical course and limited targeted therapies. Since BC cell lines have proven to be effective tools for drug discovery, we assessed the similarities between triple-negative BCs and cell lines, to identify in vitro representatives, modelling the diversity within this BC subtype. 25 BC cell lines, enriched for those lacking ER, PR and HER2 expression, were subjected to transcriptomic, genomic and epigenomic profiling analyses and comparisons were made to existing knowledge of corresponding perturbations in triple-negative BCs. Transcriptional analysis segregated ER-negative BC cell lines into three groups, displaying distinctive representations of signalling pathways and subtype-specific expression patterns. DNA copy number aberrations of triple-negative BCs were well represented in cell lines and genes with coordinately altered gene expression showed similar patterns in tumours and cell lines. Methylation events of this BC subtype were mostly retained in epigenomes of cell lines. Combined methylation and gene expression analyses revealed a subset of genes characteristic of the Claudin-low BC subtype, exhibiting epigenetic-regulated gene expression in BC cell lines and tumours, suggesting a retained mechanism of subtype-specificity. In summary, we provide a comprehensive analysis of triple-negative BC features on several molecular levels in BC cell lines, thereby creating an in-depth resource to access the suitability of individual lines as experimental models for studying BC tumour biology, biomarkers and possible therapeutic targets in the context of preclinical target validation.

1 Gene expression analysis of BCCL

Determine the files before starting the analysis and folders

```
> filePath <- "/Users/anitag/Basal.25.ESet.December.AM/Analysis_20110104/Clean_Analysis/"
> filePathStartingFiles <- paste(filePath, "startingFiles/", sep = "")
> list.files(filePathStartingFiles)
```

```
[1] "acgh.cnvs.txt"                      "all.genes.txt"
[3] "Basal.25.BeadStudioOutput.txt"      "Basal.25.Methylation.Beadstudio.txt"
[5] "Basal.25.phenoUpdated20110104.txt"  "cytobands.txt"
[7] "ensembl.55.ILMN.entrez.csv"         "full.cnvs.txt"
[9] "mirnas.txt"
```

These are libraries and in-house functions needed for analysis.

```
> library(Biobase)
> library(affy)
> library(annotate)
> library(limma)
> library(impute)
> library(lattice)
> library(grid)
> library(marray)
> library(corrgram)
> library(methylumi)
> source(paste(filePath, "R.functions/functionForBccl25Analysis.R", sep = ""))
```

Gene expression profiles were obtained from Illumina Human HWG6v2

```
> Basal.25.assembly55.lumi <- readBeads(paste(filePathStartingFiles, "Basal.25.BeadStudioOutput.txt",
+     sep = ""), ann.library = "lumiHumanAll.db", flags.on = F, p.val.thresh = 0.01)

Reading in BeadStudio output...
Perform Quality Control assessment of the LumiBatch object ...
Directly converting probe sequence to nuIDs ...
Done

Variance stabilisation normalisation...
Perform vst transformation ...
2012-05-25 10:27:56 , processing array  1
2012-05-25 10:27:56 , processing array  2
2012-05-25 10:27:56 , processing array  3
2012-05-25 10:27:56 , processing array  4
2012-05-25 10:27:56 , processing array  5
2012-05-25 10:27:57 , processing array  6
2012-05-25 10:27:57 , processing array  7
2012-05-25 10:27:57 , processing array  8
2012-05-25 10:27:57 , processing array  9
2012-05-25 10:27:58 , processing array  10
2012-05-25 10:27:58 , processing array  11
2012-05-25 10:27:58 , processing array  12
2012-05-25 10:27:58 , processing array  13
2012-05-25 10:27:59 , processing array  14
2012-05-25 10:27:59 , processing array  15
2012-05-25 10:27:59 , processing array  16
2012-05-25 10:27:59 , processing array  17
2012-05-25 10:28:00 , processing array  18
2012-05-25 10:28:00 , processing array  19
2012-05-25 10:28:00 , processing array  20
2012-05-25 10:28:01 , processing array  21
2012-05-25 10:28:01 , processing array  22
2012-05-25 10:28:01 , processing array  23
2012-05-25 10:28:01 , processing array  24
2012-05-25 10:28:01 , processing array  25
Done
```

```
Normalisation - method =  rsn
Perform rsn normalization ...
2012-05-25 10:28:05 , processing array  1
2012-05-25 10:28:05 , processing array  2
2012-05-25 10:28:05 , processing array  3
2012-05-25 10:28:06 , processing array  4
2012-05-25 10:28:06 , processing array  5
2012-05-25 10:28:07 , processing array  6
2012-05-25 10:28:07 , processing array  7
2012-05-25 10:28:07 , processing array  8
2012-05-25 10:28:07 , processing array  9
2012-05-25 10:28:07 , processing array  10
2012-05-25 10:28:08 , processing array  11
2012-05-25 10:28:08 , processing array  12
2012-05-25 10:28:08 , processing array  13
2012-05-25 10:28:09 , processing array  14
2012-05-25 10:28:09 , processing array  15
2012-05-25 10:28:09 , processing array  16
2012-05-25 10:28:10 , processing array  17
2012-05-25 10:28:10 , processing array  18
2012-05-25 10:28:10 , processing array  19
2012-05-25 10:28:11 , processing array  20
2012-05-25 10:28:11 , processing array  21
2012-05-25 10:28:11 , processing array  22
2012-05-25 10:28:12 , processing array  23
2012-05-25 10:28:12 , processing array  24
2012-05-25 10:28:12 , processing array  25
Done

Lumibatch summary
Summary of data information:
         Data File Information:


Major Operation History:
            submitted            finished
1 2012-05-25 10:27:19 2012-05-25 10:27:43
2 2012-05-25 10:27:43 2012-05-25 10:27:44
3 2012-05-25 10:27:44 2012-05-25 10:27:54
4 2012-05-25 10:27:54 2012-05-25 10:28:02
5 2012-05-25 10:28:02 2012-05-25 10:28:13

1 lumiR("/Users/anitag/Basal.25.ESet.December.AM/Analysis_20110104/Clean_Analysis/startingFiles/Basal.25
2                                                           lumiQ(x.lumi = x.lumi, detectionTh = detectio
3                                                    addNuID2lumi(x.lumi = x.lumi, lib.mapping = lib.mapp
4                                                                                                       lu
5                                                                                     lumiN(x.lumi = lumi.T
  lumiVersion
1       2.2.0
2       2.2.0
```

```
3       2.2.0
4       2.2.0
5       2.2.0

Object Information:
LumiBatch (storageMode: lockedEnvironment)
assayData: 48701 features, 25 samples
  element names: beadNum, detection, exprs, se.exprs
protocolData: none
phenoData
  sampleNames: HCC1937 HCC70 ... ZR7530 (25 total)
  varLabels: sampleID
  varMetadata: labelDescription
featureData
  featureNames: xueNZSZTNKtZCSuuBM ir43_WgJJ6COpoV7eE ... obghXh6whTR3YLoli4 (48701
    total)
  fvarLabels: ProbeID TargetID ... PROBE_COORDINATES (9 total)
  fvarMetadata: labelDescription
experimentData: use 'experimentData(object)'
Annotation: lumiHumanAll.db
Control Data: Available
QC information: Please run summary(x, 'QC') for details!
QC summary
Data dimension:  48701 genes x 25 samples

Summary of Samples:
                          HCC1937   HCC70 MDAMB468   SUM159  HCC1428  SUM149 HCC1954 MDAMB231
mean                       6.7930  6.5340   6.5260   6.4070   6.4860  6.4720  6.4780   6.5080
standard deviation         1.6810  1.5860   1.5570   1.4690   1.5550  1.5120  1.5670   1.6020
detection rate(0.01)       0.3147  0.2562   0.2886   0.2633   0.2722  0.2553  0.2643   0.2723
distance to sample mean  106.9000 97.6500  91.0500 106.6000 105.9000 95.2700 88.5300 100.1000
                            BT483 MDAMB157   HS578T MDAMB436 HCC1143    HCC38  HCC1187  HCC1569
mean                       6.4180   6.6470   6.6190   6.5600  6.6610   6.6810   6.5000   6.5800
standard deviation         1.5370   1.5570   1.5380   1.4870  1.6210   1.6380   1.5480   1.6150
detection rate(0.01)       0.2696   0.2838   0.2737   0.2775  0.2893   0.3009   0.2717   0.2864
distance to sample mean  104.3000 105.0000 90.6100  99.6700 92.4700 101.3000 107.6000 104.4000
                            BT549  SUM1315   SUM225   SUM190     T47D   BT474     BT20    SKBR3
mean                       6.6190   6.5190   6.5310   6.5980   6.5870  6.4940   6.7070   7.1190
standard deviation         1.6430   1.5600   1.5530   1.6580   1.6220  1.5450   1.6620   1.3130
detection rate(0.01)       0.2751   0.2753   0.2741   0.2595   0.2672  0.2653   0.2992   0.2419
distance to sample mean  105.2000 104.6000 91.2000 107.5000 101.4000 97.8200 102.9000 155.9000
                           ZR7530
mean                       7.0490
standard deviation         1.2550
detection rate(0.01)       0.2356
distance to sample mean  152.7000

Major Operation History:
            submitted            finished
1 2012-05-25 10:27:19 2012-05-25 10:27:43
```

```
2 2012-05-25 10:27:43 2012-05-25 10:27:44

1 lumiR("/Users/anitag/Basal.25.ESet.December.AM/Analysis_20110104/Clean_Analysis/startingFiles/Basal.25
2                                                            lumiQ(x.lumi = x.lumi, detectionTh = detectio
  lumiVersion
1       2.2.0
2       2.2.0
0 Duplicated sequences
Retrieving TRANSCRIPT IDs...Done
Retrieving PROBE IDs...Done
Retrieving mapping positions...Done
Retrieving genenames...Done
Retrieving gene symbols...Done
Retrieving entrezIDs...Done
Retrieving accession numbers...Done
Retrieving Ensembl IDs...Done
Retrieving Unigene IDs...Done
Retrieving Cytobands...Done
```

Since the annotation of Illumina probes obtained by the lumiHumanAll.db package is limited, we extended the mapping of Illumina probes by downloading Illumina probe annotation via Ensembl 55 /Biomart. This resulted in a file (called ensembl.55.ILMN.txt) comprising of 25796 Ensembl genes with Illumina identifiers.

```
> ensembl.55.ILMN <- read.csv(paste(filePathStartingFiles, "ensembl.55.ILMN.entrez.csv",
+     sep = ""), sep = ",", stringsAsFactors = F, na.strings = c("", " ", "NA",
+     "#N/A"))
> names(ensembl.55.ILMN) <- c("ensg", "description", "chrom", "start", "end",
+     "symbol", "status", "entrez", "ILMN")
> names(ensembl.55.ILMN)

[1] "ensg"        "description" "chrom"       "start"       "end"         "symbol"
[7] "status"      "entrez"      "ILMN"

> names(fData(Basal.25.assembly55.lumi))

 [1] "ProbeID"               "TargetID"              "SYMBOL"                "CHROMOSOME"
 [5] "DEFINITION"            "ACCESSION"             "PROBE_START"           "PROBE_CHR_ORIENTATION"
 [9] "PROBE_COORDINATES"     "TRANSCRIPT"            "probeID"               "chrom"
[13] "start"                 "end"                   "description"           "symbol"
[17] "entrez"                "refseq"                "ensg"                  "unigene"
[21] "cytoband"

> sum(is.element(fData(Basal.25.assembly55.lumi)$probeID, ensembl.55.ILMN$ILMN))

[1] 23500

> Basal.25.lumi.assembly55.probes <- ensembl.55.ILMN[match(fData(Basal.25.assembly55.lumi)$probeID,
+     ensembl.55.ILMN$ILMN, nomatch = NA), ]
> Basal.25.lumi.assembly55.probes.R.ensg <- ensembl.55.ILMN[match(fData(Basal.25.assembly55.lumi)$ensg,
+     ensembl.55.ILMN$ensg, nomatch = NA), ]
> nrow(Basal.25.lumi.assembly55.probes)
```

```
[1] 48701

> nrow(Basal.25.lumi.assembly55.probes.R.ensg)

[1] 48701

> Basal.25.lumi.assembly55.probes[is.na(Basal.25.lumi.assembly55.probes$ensg),
+     ] <- Basal.25.lumi.assembly55.probes.R.ensg[is.na(Basal.25.lumi.assembly55.probes$ensg),
+     ]
> Basal.25.lumi.assembly55.probes$chrom[Basal.25.lumi.assembly55.probes$chrom ==
+     "X"] <- 23
> Basal.25.lumi.assembly55.probes$chrom[Basal.25.lumi.assembly55.probes$chrom ==
+     "Y"] <- 24
> Basal.25.lumi.assembly55.probes$chrom <- as.integer(Basal.25.lumi.assembly55.probes$chrom)
> sum(is.element(Basal.25.lumi.assembly55.probes$chrom, 1:24))

[1] 25805

> unique(Basal.25.lumi.assembly55.probes$chrom)

 [1] 19 12  7  6  9 NA  1  4 22 16 17  3  2  8 10  5 11 23 15 24 20 14 21 13 18

> fData(Basal.25.assembly55.lumi)$nuID <- Basal.25.lumi.assembly55.probes$nuID
> fData(Basal.25.assembly55.lumi)$ILMN <- Basal.25.lumi.assembly55.probes$probeID
> fData(Basal.25.assembly55.lumi)$ensg <- Basal.25.lumi.assembly55.probes$ensg
> fData(Basal.25.assembly55.lumi)$symbol <- Basal.25.lumi.assembly55.probes$symbol
> fData(Basal.25.assembly55.lumi)$description <- Basal.25.lumi.assembly55.probes$description
> fData(Basal.25.assembly55.lumi)$chrom <- Basal.25.lumi.assembly55.probes$chrom
> fData(Basal.25.assembly55.lumi)$start <- Basal.25.lumi.assembly55.probes$start
> fData(Basal.25.assembly55.lumi)$end <- Basal.25.lumi.assembly55.probes$end
> fData(Basal.25.assembly55.lumi)$status <- Basal.25.lumi.assembly55.probes$status
> fData(Basal.25.assembly55.lumi)$entrez <- Basal.25.lumi.assembly55.probes$entrez
> head(fData(Basal.25.assembly55.lumi), 5)

                     ProbeID TargetID SYMBOL CHROMOSOME
xueNZSZTNKtZCSuuBM 2140735     RPS9   RPS9
ir43_WgJJ6COpoV7eE 6550370      UBC    UBC
97viJ90hzUX_rZ.nvY 2690379   EEF1A1 EEF1A1          6
udI0NuTXy4H3vtdCTo 4590356     TUBB   TUBB
ckjYiQh5.0oJfoZ5K4 4260048      TXN    TXN
                                                                                  DEFINITION
xueNZSZTNKtZCSuuBM                               Homo sapiens ribosomal protein S9 (RPS9), mRNA.
ir43_WgJJ6COpoV7eE                                         Homo sapiens ubiquitin C (UBC), mRNA.
97viJ90hzUX_rZ.nvY Homo sapiens eukaryotic translation elongation factor 1 alpha 1 (EEF1A1), mRNA.
udI0NuTXy4H3vtdCTo                          Homo sapiens tubulin, beta polypeptide (TUBB), mRNA.
ckjYiQh5.0oJfoZ5K4                                         Homo sapiens thioredoxin (TXN), mRNA.
                     ACCESSION PROBE_START PROBE_CHR_ORIENTATION
xueNZSZTNKtZCSuuBM NM_001013.2         416
ir43_WgJJ6COpoV7eE NM_021009.1        1856
97viJ90hzUX_rZ.nvY NM_001402.4        1293                     -
udI0NuTXy4H3vtdCTo NM_001069.1        1408
```

```
ckjYiQh5.0oJfoZ5K4 NM_003329.1          72
                                       PROBE_COORDINATES  TRANSCRIPT      probeID chrom     start
xueNZSZTNKtZCSuuBM                                       GI_14141192 ILMN_1343289    19  54704726
ir43_WgJJ6COpoV7eE                                       GI_20149305 ILMN_1343290    12 125396194
97viJ90hzUX_rZ.nvY 74284362-74284378:74284474-74284506 GI_25453469 ILMN_1343291     7  22459063
udI0NuTXy4H3vtdCTo                                        GI_4507728 ILMN_1343292     6   3153919
ckjYiQh5.0oJfoZ5K4                                        GI_4507744 ILMN_1343293     9 113006300
                         end
xueNZSZTNKtZCSuuBM  54752862
ir43_WgJJ6COpoV7eE 125399577
97viJ90hzUX_rZ.nvY  22672544
udI0NuTXy4H3vtdCTo   3157809
ckjYiQh5.0oJfoZ5K4 113018821

xueNZSZTNKtZCSuuBM                                                                  40S ribosomal protein S
ir43_WgJJ6COpoV7eE                                                                                 Ubiquiti
97viJ90hzUX_rZ.nvY                                                             STEAP family protein MGC8704
udI0NuTXy4H3vtdCTo                                                                       Tubulin beta-2A chai
ckjYiQh5.0oJfoZ5K4 Thioredoxin (Trx)(ATL-derived factor)(ADF)(Surface-associated sulphydryl protein)(SAS
                       symbol entrez    refseq            ensg   unigene cytoband status
xueNZSZTNKtZCSuuBM       RPS9     NA NM_001013 ENSG00000170889 Hs.467284  19q13.4  KNOWN
ir43_WgJJ6COpoV7eE        UBC   7316 NM_021009 ENSG00000150991 Hs.520348  12q24.3  KNOWN
97viJ90hzUX_rZ.nvY AC099759.1     NA NM_001402 ENSG00000105889 Hs.520703   6q14.1  KNOWN
udI0NuTXy4H3vtdCTo     TUBB2A   7280 NM_001069 ENSG00000137267 Hs.654543     6p25  KNOWN
ckjYiQh5.0oJfoZ5K4        TXN   7295 NM_003329 ENSG00000136810 Hs.435136     9q31  KNOWN
```

Add the clinicopathological information provided as Supplementary Table 1 to the expressionSet and export all relevant tables

```
> Basal.25.assembly55.lumi <- readPheno(Basal.25.assembly55.lumi, paste(filePathStartingFiles,
+     "Basal.25.phenoUpdated20110104.txt", sep = ""))
```

```
Reading pheno file
25 Pheno sampleNames
25 Eset sampleNames
25 Matching sampleNames
 HCC1937 HCC70 MDAMB468 SUM159 HCC1428 SUM149 HCC1954 MDAMB231 BT483 MDAMB157 HS578T MDAMB436 HCC1143 HC
Done
```

```
> spitTables(Basal.25.assembly55.lumi, output.directory = "BCCL.expression", project = "BCCL25")
```

```
writing featureData table
Writing phenoData table
Writing beadNum table
Writing detection table
Writing exprs table
Writing flags table
Writing se.exprs table
Done
```

Reduce the data to variable probe-sets and to one probe-set per gene

```
> Basal.25.assembly55.lumi.mapped <- removeUnmappedProbes(Basal.25.assembly55.lumi)
```

```
Removing unmapped probes
 25805 mapped probes remaining
 Done
```

```
> Basal.25.assembly55.lumi.flagged <- removeProbesByFlags(Basal.25.assembly55.lumi.mapped,
+     flag.limit = 20)
```

```
Removing probes with 20 flags or more
 13460 probes remaining
 Done
```

```
> Basal.25.assembly55.lumi.MAD <- removeProbesByMAD(Basal.25.assembly55.lumi.flagged,
+     mad.limit = 0.4)
```

```
 6151 probes with a median absolute deviation greater than 0.4
 Done
```

```
> Basal.25.assembly55.lumi.MAD <- removeReplicateProbesByMAD(Basal.25.assembly55.lumi.MAD,
+     "ensg")
```

```
 5693 probes remaining
 Done
```

```
> Basal.25.assembly55.lumi.MAD.c <- centerGenes(Basal.25.assembly55.lumi.MAD,
+     center = "mean")
> validObject(Basal.25.assembly55.lumi.MAD)
```

```
[1] TRUE
```

Hierarchical Clustering of gene expression profiles and make the phenoBar for Figure 1

```
> Basal.25.assembly55.lumi.MAD.c <- Basal.25.assembly55.lumi.MAD.c[, order(Basal.25.assembly55.lumi.MAD.c
+     decreasing = F)]
> pData(Basal.25.assembly55.lumi.MAD.c)$expression.cluster2 <- as.character(pData(Basal.25.assembly55.lum
> pData(Basal.25.assembly55.lumi.MAD.c)$expression.cluster2[grep("Luminal", pData(Basal.25.assembly55.lum
> pData(Basal.25.assembly55.lumi.MAD.c)$expression.cluster2[grep("BasalA", pData(Basal.25.assembly55.lumi
> pData(Basal.25.assembly55.lumi.MAD.c)$expression.cluster2[grep("BasalB", pData(Basal.25.assembly55.lumi
> bw2 <- c("black", "darkgrey", "black", "darkgrey", "darkgrey", "black")
> plotPhenoBar(Basal.25.assembly55.lumi.MAD.c, cluster = NULL, phenotypes = pData(Basal.25.assembly55.lum
+     c(8, 7, 5)], pheno.colours = bw2, device = "PDF", project = "BCCLmeanExpressionCluster")
```

```
pdf
  2
```

2 Investigation of publicly available gene signatures in each cell line

```
> setwd("GeneSignatureAnalysis")
> pData(Basal.25.assembly55.lumi.flagged)$expression.cluster2 <- as.character(pData(Basal.25.assembly55.l
```

```
> pData(Basal.25.assembly55.lumi.flagged)$expression.cluster2[grep("Luminal",
+     pData(Basal.25.assembly55.lumi.flagged)$expression.cluster2)] <- "Cluster1"
> pData(Basal.25.assembly55.lumi.flagged)$expression.cluster2[grep("BasalA", pData(Basal.25.assembly55.lu
> pData(Basal.25.assembly55.lumi.flagged)$expression.cluster2[grep("BasalB", pData(Basal.25.assembly55.lu
> Basal.25.assembly55.lumi.c <- centerGenes(Basal.25.assembly55.lumi.flagged,
+     center = "mean")
> Basal.25.assembly55.lumi.c <- weightedAverageSignature(Basal.25.assembly55.lumi.c,
+     Basal.25.assembly55.lumi.c$expression.cluster2, gene.list.file = "GeneSignatureList/HoadleyCluster
+     gene.list.metric = "UpDown", eset.id = "ensg", project = "Hoadley.EGFR.Cluster1.ensg",
+     pheno.colours = c("red", "orange", "blue"), main = "Hoadley EGFR", return.eset = T,
+     eset.pheno.label = "EGFR.Cluster1")

32 matching identifiers in Gene List and Expression Set

> Basal.25.assembly55.lumi.c <- weightedAverageSignature(Basal.25.assembly55.lumi.c,
+     Basal.25.assembly55.lumi.c$expression.cluster2, gene.list.file = "GeneSignatureList/HoadleyCluster2
+     gene.list.metric = "UpDown", eset.id = "ensg", project = "Hoadley.EGFR.Cluster2.ensg",
+     pheno.colours = c("red", "orange", "blue"), main = "Hoadley EGFR", return.eset = T,
+     eset.pheno.label = "EGFR.Cluster2")

24 matching identifiers in Gene List and Expression Set

> Basal.25.assembly55.lumi.c <- weightedAverageSignature(Basal.25.assembly55.lumi.c,
+     Basal.25.assembly55.lumi.c$expression.cluster2, gene.list.file = "GeneSignatureList/HoadleyCluster3
+     gene.list.metric = "UpDown", eset.id = "ensg", project = "Hoadley.EGFR.Cluster3.ensg",
+     pheno.colours = c("red", "orange", "blue"), main = "Hoadley EGFR", return.eset = T,
+     eset.pheno.label = "EGFR.Cluster3")

127 matching identifiers in Gene List and Expression Set

> Hoadley.EGFR.Cluster1.KW.p <- kruskal.test(Basal.25.assembly55.lumi.c$EGFR.Cluster1 ~
+     as.factor(Basal.25.assembly55.lumi.c$expression.cluster2))$p.value
> Hoadley.EGFR.Cluster2.KW.p <- kruskal.test(Basal.25.assembly55.lumi.c$EGFR.Cluster2 ~
+     as.factor(Basal.25.assembly55.lumi.c$expression.cluster2))$p.value
> Hoadley.EGFR.Cluster3.KW.p <- kruskal.test(Basal.25.assembly55.lumi.c$EGFR.Cluster3 ~
+     as.factor(Basal.25.assembly55.lumi.c$expression.cluster2))$p.value
> Basal.25.assembly55.lumi.c <- weightedAverageSignature(Basal.25.assembly55.lumi.c,
+     Basal.25.assembly55.lumi.c$expression.cluster2, project = "MET.ShortList",
+     gene.list = "GeneSignatureList/MET_signature_mapped_to_human2.txt", main = "MET short list signatu
+     pheno.colours = c("red", "orange", "blue"), eset.id = "entrez", eset.pheno.label = "MET.short.signa
+     gene.list.metric = "UpDownNumeric", return.eset = T)

213 matching identifiers in Gene List and Expression Set

> MET.KW.p <- kruskal.test(Basal.25.assembly55.lumi.c$MET.short.signature ~ as.factor(Basal.25.assembly5
> Basal.25.assembly55.lumi.c <- weightedAverageSignature(Basal.25.assembly55.lumi.c,
+     Basal.25.assembly55.lumi.c$expression.cluster2, project = "StromaRelatedGenes",
+     gene.list = "GeneSignatureList/StromalRelatedGenes_West_PlosBiol2005BMCGenomics2007_numeric.txt",
+     main = "Stroma signature in BCCL Data", pheno.colours = c("red", "orange",
+         "blue"), eset.id = "entrez", eset.pheno.label = "Stroma.signature",
+     gene.list.metric = "UpDown", return.eset = T)
```

171 matching identifiers in Gene List and Expression Set

```
> Stroma.KW.p <- kruskal.test(Basal.25.assembly55.lumi.c$Stroma.signature ~ as.factor(Basal.25.assembly55
> Basal.25.assembly55.lumi.c <- weightedAverageSignature(Basal.25.assembly55.lumi.c,
+     Basal.25.assembly55.lumi.c$expression.cluster2, project = "MammosphereRelatedGenes",
+     gene.list = "GeneSignatureList/MammosphereSign_Creighton_PNAS2009_numeric.txt",
+     main = "Mammosphere signature in BCCL Data", pheno.colours = c("red", "orange",
+         "blue"), eset.id = "entrez", eset.pheno.label = "Mammosphere.signature",
+     gene.list.metric = "UpDown", return.eset = T)
```

192 matching identifiers in Gene List and Expression Set

```
> Mammosphere.KW.p <- kruskal.test(Basal.25.assembly55.lumi.c$Mammosphere.signature ~
+     as.factor(Basal.25.assembly55.lumi.c$expression.cluster2))$p.value
> Basal.25.assembly55.lumi.c <- weightedAverageSignature(Basal.25.assembly55.lumi.c,
+     Basal.25.assembly55.lumi.c$expression.cluster2, project = "CD24CD44_RelatedGenes",
+     gene.list = "GeneSignatureList/MammosphereSign_Shipitsin_CancerCell2007_numeric.txt",
+     main = "CD24-CD44 signature in BCCL Data", pheno.colours = c("red", "orange",
+         "blue"), eset.id = "entrez", eset.pheno.label = "CD24.CD44.signature",
+     gene.list.metric = "UpDown", return.eset = T)
```

394 matching identifiers in Gene List and Expression Set

```
> CD24.CD44.KW.p <- kruskal.test(Basal.25.assembly55.lumi.c$CD24.CD44.signature ~
+     as.factor(Basal.25.assembly55.lumi.c$expression.cluster2))$p.value
> Basal.25.assembly55.lumi.c <- weightedAverageSignature(Basal.25.assembly55.lumi.c,
+     Basal.25.assembly55.lumi.c$expression.cluster2, project = "Apocrine.Basal",
+     gene.list = "GeneSignatureList/Apocrine_Farmer_Oncogene2005.txt", main = "Apocrine.basal signature
+     pheno.colours = c("red", "orange", "blue"), eset.id = "entrez", eset.pheno.label = "Apocrine.basal
+     gene.list.metric = "UpDown", return.eset = T)
```

9 matching identifiers in Gene List and Expression Set

```
> Apocrine.KW.p <- kruskal.test(Basal.25.assembly55.lumi.c$Apocrine.basal.signature ~
+     as.factor(Basal.25.assembly55.lumi.c$expression.cluster2))$p.value
> Basal.25.assembly55.lumi.c <- weightedAverageSignature(Basal.25.assembly55.lumi.c,
+     Basal.25.assembly55.lumi.c$expression.cluster2, project = "Interferon.relatedGenes",
+     gene.list = "GeneSignatureList/InterferonRegulatedGenes_Hu_BMCGenomics2006.txt",
+     main = "Interferon-regulated genes in BCCL Data", pheno.colours = c("red",
+         "orange", "blue"), eset.id = "entrez", eset.pheno.label = "Interferon.signature",
+     gene.list.metric = "UpDown", return.eset = T)
```

6 matching identifiers in Gene List and Expression Set

```
> Interferon.KW.p <- kruskal.test(Basal.25.assembly55.lumi.c$Interferon.signature ~
+     as.factor(Basal.25.assembly55.lumi.c$expression.cluster2))$p.value
> Basal.25.assembly55.lumi.c <- weightedAverageSignature(Basal.25.assembly55.lumi.c,
+     Basal.25.assembly55.lumi.c$expression.cluster2, project = "IGF1.relatedGenes",
+     gene.list = "GeneSignatureList/IGF1regulatedGenes_Creighton_JCO2008.txt",
+     main = "IGF1 genes in BCCL Data", pheno.colours = c("red", "orange", "blue"),
+     eset.id = "entrez", eset.pheno.label = "IGF1.signature", gene.list.metric = "UpDown",
+     return.eset = T)
```

```
510 matching identifiers in Gene List and Expression Set

> IGF1.KW.p <- kruskal.test(Basal.25.assembly55.lumi.c$IGF1.signature ~ as.factor(Basal.25.assembly55.lum
> Basal.25.assembly55.lumi.c <- weightedAverageSignature(Basal.25.assembly55.lumi.c,
+     Basal.25.assembly55.lumi.c$expression.cluster2, project = "Natrajan.basal",
+     gene.list = "GeneSignatureList/Natrajan.basal.signature.txt", main = "Natrajan Basal genes in BCCL
+     pheno.colours = c("red", "orange", "blue"), eset.id = "ensg", eset.pheno.label = "Natrajan.basal",
+     gene.list.metric = "UpDown", return.eset = T)

188 matching identifiers in Gene List and Expression Set

> Natrajan.basal.KW.p <- kruskal.test(Basal.25.assembly55.lumi.c$Natrajan.basal ~
+     as.factor(Basal.25.assembly55.lumi.c$expression.cluster2))$p.value
> Basal.25.assembly55.lumi.c <- weightedAverageSignature(Basal.25.assembly55.lumi.c,
+     Basal.25.assembly55.lumi.c$expression.cluster2, project = "Cordenonsi",
+     gene.list = "GeneSignatureList/CordenonsiCell2011_SuppTable1.txt", main = "G3 v G1 genes in BCCL Da
+     pheno.colours = c("red", "orange", "blue"), eset.id = "symbol", eset.pheno.label = "Cordenonsi",
+     gene.list.metric = "UpDown", return.eset = T)

60 matching identifiers in Gene List and Expression Set

> Cordensoni.KW.p <- kruskal.test(Basal.25.assembly55.lumi.c$Cordenonsi ~ as.factor(Basal.25.assembly55.l
```

This figure makes Supplementary Figure 1

```
> pdf("CorrelationOfGeneSignaturesInBccls.pdf")
> corrgram(as.data.frame(pData(Basal.25.assembly55.lumi.c)[, -1:-20]), order = TRUE,
+     lower.panel = panel.shade, upper.panel = panel.pie, main = "Correlation of Gene Signatures in Bccls
+     cex.labels = 0.4)
> dev.off()

pdf
  2

> BCCL25.pvals <- data.frame(EGFR.Cluster2 = Hoadley.EGFR.Cluster2.KW.p, MET = MET.KW.p,
+     Stroma = Stroma.KW.p, Mammosphere = Mammosphere.KW.p, CD24.CD44 = CD24.CD44.KW.p,
+     Apocrine = Apocrine.KW.p, Interferon = Interferon.KW.p, IGF1 = IGF1.KW.p,
+     Basal.tumour = Natrajan.basal.KW.p, Cordensoni.KW.p)
> BCCL25.sig.values <- pData(Basal.25.assembly55.lumi.c)[, c(25, 26, 27, 24, 22,
+     30, 29, 28, 31, 32)]
> names(BCCL25.sig.values) <- c("Stroma", "Mammosphere", "CD24.CD44", "MET", "EGFR",
+     "IGF1", "Interferon", "Apocrine", "TNBC.Grade3.tumour", "Grade3v1")
> BCCL25.sig.ranks <- apply(BCCL25.sig.values, 2, rank)
> BCCL25.sig.ranks <- data.frame(sampleNames = sampleNames(Basal.25.assembly55.lumi.c),
+     BCCL25.sig.ranks)
> write.table(BCCL25.sig.ranks, "BCCL25.sig.ranks.txt", sep = "\t", row.names = F)
> BCCL25.sig <- Basal.25.assembly55.lumi.c
> BCCL25.sig <- BCCL25.sig[, order(BCCL25.sig$cluster.order)]
> BCCL25.sig <- readPheno(BCCL25.sig, "BCCL25.sig.ranks.txt")

Reading pheno file
25 Pheno sampleNames
```

```
25 Eset sampleNames
25 Matching sampleNames
 BT474 T47D HCC1428 BT483 ZR7530 SKBR3 SUM190 SUM225 HS578T MDAMB157 BT549 SUM1315 MDAMB436 SUM159 MDAMB2
Done
```

```
> sig.pcs <- maPalette(low = "white", high = "darkblue", k = 25)
> pal <- maPalette(low = colors()[29], high = colors()[554], mid = "white", k = 27)
> plotPhenoBar(BCCL25.sig, cluster = NULL, phenotypes = pData(BCCL25.sig), pheno.colours = sig.pcs,
+     plot.legend = F, device = "PDF", project = "BCCL.signature")
```

```
pdf
  2
```

```
> pdf("ColorBar.pdf")
> maColorBar(seq(1, 25, 1), col = sig.pcs, horizontal = TRUE)
> dev.off()
```

```
pdf
  2
```

return to the working folder

```
> setwd("..")
> setwd("..")
```

3 Investigation of publicly available gene signatures in each cell line - based on centroid classification

```
> setwd(paste(filePath, "BreastTumourClassCentroids/", sep = ""))
> Basal.25.assembly55.lumi.c <- centroidCorrelation(Basal.25.assembly55.lumi.c,
+     "PAM50.centroids.txt", centroids = 5, centroid.id = "ensg", eset.id = "ensg",
+     project = "BCCL.PAM50.ensg", cor.method = "spearman", return.eset = T, pheno.label = "PAM50.ensg.ne
```

```
46 matching identifiers
```

```
> Basal.25.assembly55.lumi.c <- centroidCorrelation(Basal.25.assembly55.lumi.c,
+     "PAM50.No.Normal.centroids.txt", centroids = 4, centroid.id = "ensg", eset.id = "ensg",
+     project = "BCCL.PAM50.No.Normal.ensg", cor.method = "spearman", return.eset = T,
+     pheno.label = "PAM50.No.Normal.ensg.nearest.centroid")
```

```
46 matching identifiers
```

```
> Basal.25.assembly55.lumi.c <- centroidCorrelation(Basal.25.assembly55.lumi.c,
+     "Sorlie500.centroids.txt", centroids = 5, centroid.id = "ensg", eset.id = "ensg",
+     project = "BCCL25.Sorlie500.ensg", cor.method = "pearson", return.eset = T,
+     pheno.label = "Sorlie500.ensg.nearest.centroid")
```

```
364 matching identifiers
```

```
> Basal.25.assembly55.lumi.c <- centroidCorrelation(Basal.25.assembly55.lumi.c,
+     "Hu306.centroids.txt", centroids = 5, centroid.id = "ensg", eset.id = "ensg",
+     project = "BCCL25.Hu306.ensg", cor.method = "spearman", return.eset = T,
+     pheno.label = "Hu306.ensg.nearest.centroid")

270 matching identifiers

> Basal.25.assembly55.lumi.c <- centroidCorrelation(Basal.25.assembly55.lumi.c,
+     "ClaudinLow.centroids.txt", centroids = 2, centroid.id = "symbol", eset.id = "symbol",
+     project = "BCCL25.ClaudinLow.gene.symbol", cor.method = "spearman", return.eset = T,
+     pheno.label = "ClaudinLow.symbol.nearest.centroid")

648 matching identifiers

> Basal.25.assembly55.lumi.c <- centroidCorrelation(Basal.25.assembly55.lumi.c,
+     "Teschendorff.centroids.txt", centroids = 5, centroid.id = "symbol", eset.id = "symbol",
+     project = "BCCL25.Teschendorff.ensg", cor.method = "spearman", return.eset = T,
+     pheno.label = "Teschendorff.ensg.nearest.centroid")

583 matching identifiers

> Basal.25.assembly55.lumi.c <- centroidCorrelation(Basal.25.assembly55.lumi.c,
+     "NKI295.70.gene.centroids.txt", centroids = 2, centroid.id = "ensg", eset.id = "ensg",
+     project = "BCCL25.NKI295.70.gene.ensg", cor.method = "spearman", return.eset = T,
+     pheno.label = "NKI295.70.gene.ensg.nearest.centroid")

44 matching identifiers

> Basal.25.assembly55.lumi.c <- centroidCorrelation(Basal.25.assembly55.lumi.c,
+     "NKI295.Wound.healing.centroids.txt", centroids = 2, centroid.id = "ensg",
+     eset.id = "ensg", project = "BCCL25.Wound.healing.gene.ensg", cor.method = "spearman",
+     return.eset = T, pheno.label = "NKI295.Wound.healing.ensg.nearest.centroid")

297 matching identifiers

> setwd("..")
```

4 Analysis of aCGH profiles of BCCL

Change to working directory for genomic analysis, establish the genomic aberrations

```
> setwd(paste(filePath, "BCCL.genomicAnalysis/GPR", sep = ""))
> basal25cgh.raw <- dietCGH(design.file = "Basal.25.design.rep.txt", fdata.file = "Ann32K.NCBI37.55.txt",
+     rhm = T, MAD = 1)

Design file chosen was "Basal.25.design.rep.txt"
Checking directory for results files...OK
Reading results files
Reading results file 1 32500871.gpr 34272 probes
Reading results file 2 32500870.gpr 34272 probes
Reading results file 3 32502925.gpr 34272 probes
```

```
Reading results file 4 32500862.gpr 34272 probes
Reading results file 5 32500854.gpr 34272 probes
Reading results file 6 32500858.gpr 34272 probes
Reading results file 7 32500860.gpr 34272 probes
Reading results file 8 32500846.gpr 34272 probes
Reading results file 9 32500861.gpr 34272 probes
Reading results file 10 32500876.gpr 34272 probes
Reading results file 11 32500849.gpr 34272 probes
Reading results file 12 32500859.gpr 34272 probes
Reading results file 13 32500842.gpr 34272 probes
Reading results file 14 32500855.gpr 34272 probes
Reading results file 15 32500853.gpr 34272 probes
Reading results file 16 32500850.gpr 34272 probes
Reading results file 17 32500857.gpr 34272 probes
Reading results file 18 32500844.gpr 34272 probes
Reading results file 19 32500867.gpr 34272 probes
Reading results file 20 32500864.gpr 34272 probes
Reading results file 21 32500848.gpr 34272 probes
Reading results file 22 32500845.gpr 34272 probes
Reading results file 23 32500866.gpr 34272 probes
Reading results file 24 32500877.gpr 34272 probes
Reading results file 25 32500869.gpr 34272 probes
Reading results file 26 32500851.gpr 34272 probes
Assigning flags...Done
Converting raw data to MA...Done
valid BACE.cgh Object
annotating ExpressionSet CGH
31619 annotated probes
Done
Normalising by Block
1 BT20
2 BT474
3 BT474.1
4 BT483
5 BT549
6 HCC1143
7 HCC1187
8 HCC1428
9 HCC1569
10 HCC1937
11 HCC1954
12 HCC38
13 HCC70
14 HS578T
15 MDAMB157
16 MDAMB231
17 MDAMB436
18 MDAMB468
19 SKBR3
20 SUM1315
```

```
21 SUM149
22 SUM159
23 SUM190
24 SUM225
25 T47D
26 ZR7530
Done
Combining 25 replicates
Removing probes with a 1 MAD deviation from the running median of 3
1 BT20
2 BT474
3 BT483
4 BT549
5 HCC1143
6 HCC1187
7 HCC1428
8 HCC1569
9 HCC1937
10 HCC1954
11 HCC38
12 HCC70
13 HS578T
14 MDAMB157
15 MDAMB231
16 MDAMB436
17 MDAMB468
18 SKBR3
19 SUM1315
20 SUM149
21 SUM159
22 SUM190
23 SUM225
24 T47D
25 ZR7530
Recalculating MADs...Done
Imputing missing values
1         2         3         4         5         6         7         8         9         10         11
1         2         3         4         5         6         7         8         9         10         11
1         2         3         4         5         6         7         8         9         10         11
1         2         3         4         5         6         7         8         9         10         11
1         2         3         4         5         6         7         8         9         10         11
1         2         3         4         5         6         7         8         9         10         11
1         2         3         4         5         6         7         8         9         10         11
1         2         3         4         5         6         7         8         9         10         11
1         2         3         4         5         6         7         8         9         10         11
1         2         3         4         5         6         7         8         9         10         11
1         2         3         4         5         6         7         8         9         10         11
1         2         3         4         5         6         7         8         9         10         11
1         2         3         4         5         6         7         8         9         10         11
1         2         3         4         5         6         7         8         9         10         11
```

```
1         2         3         4         5         6         7         8         9         10         11
1         2         3         4         5         6         7         8         9         10         11
1         2         3         4         5         6         7         8         9         10         11
1         2         3         4         5         6         7         8         9         10         11
1         2         3         4         5         6         7         8         9         10         11
1         2         3         4         5         6         7         8         9         10         11
1         2         3         4         5         6         7         8         9         10         11
1         2         3         4         5         6         7         8         9         10         11
1         2         3         4         5         6         7         8         9         10         11
1         2         3         4         5         6         7         8         9         10         11
1         2         3         4         5         6         7         8         9         10         11
Done
```

```
> basal25cbs <- cbsCGH(basal25cgh.raw)
```

```
Smoothing data using cbs
Analyzing: cbs.1
  current chromosome: 1
  current chromosome: 2
  current chromosome: 3
  current chromosome: 4
  current chromosome: 5
  current chromosome: 6
  current chromosome: 7
  current chromosome: 8
  current chromosome: 9
  current chromosome: 10
  current chromosome: 11
  current chromosome: 12
  current chromosome: 13
  current chromosome: 14
  current chromosome: 15
  current chromosome: 16
  current chromosome: 17
  current chromosome: 18
  current chromosome: 19
  current chromosome: 20
  current chromosome: 21
  current chromosome: 22
  current chromosome: 23
  current chromosome: 24
Analyzing: cbs.2
  current chromosome: 1
  current chromosome: 2
  current chromosome: 3
  current chromosome: 4
  current chromosome: 5
  current chromosome: 6
  current chromosome: 7
  current chromosome: 8
```

```
  current chromosome: 9
  current chromosome: 10
  current chromosome: 11
  current chromosome: 12
  current chromosome: 13
  current chromosome: 14
  current chromosome: 15
  current chromosome: 16
  current chromosome: 17
  current chromosome: 18
  current chromosome: 19
  current chromosome: 20
  current chromosome: 21
  current chromosome: 22
  current chromosome: 23
  current chromosome: 24
Analyzing: cbs.3
  current chromosome: 1
  current chromosome: 2
  current chromosome: 3
  current chromosome: 4
  current chromosome: 5
  current chromosome: 6
  current chromosome: 7
  current chromosome: 8
  current chromosome: 9
  current chromosome: 10
  current chromosome: 11
  current chromosome: 12
  current chromosome: 13
  current chromosome: 14
  current chromosome: 15
  current chromosome: 16
  current chromosome: 17
  current chromosome: 18
  current chromosome: 19
  current chromosome: 20
  current chromosome: 21
  current chromosome: 22
  current chromosome: 23
  current chromosome: 24
Analyzing: cbs.4
  current chromosome: 1
  current chromosome: 2
  current chromosome: 3
  current chromosome: 4
  current chromosome: 5
  current chromosome: 6
  current chromosome: 7
  current chromosome: 8
```

```
  current chromosome: 9
  current chromosome: 10
  current chromosome: 11
  current chromosome: 12
  current chromosome: 13
  current chromosome: 14
  current chromosome: 15
  current chromosome: 16
  current chromosome: 17
  current chromosome: 18
  current chromosome: 19
  current chromosome: 20
  current chromosome: 21
  current chromosome: 22
  current chromosome: 23
  current chromosome: 24
Analyzing: cbs.5
  current chromosome: 1
  current chromosome: 2
  current chromosome: 3
  current chromosome: 4
  current chromosome: 5
  current chromosome: 6
  current chromosome: 7
  current chromosome: 8
  current chromosome: 9
  current chromosome: 10
  current chromosome: 11
  current chromosome: 12
  current chromosome: 13
  current chromosome: 14
  current chromosome: 15
  current chromosome: 16
  current chromosome: 17
  current chromosome: 18
  current chromosome: 19
  current chromosome: 20
  current chromosome: 21
  current chromosome: 22
  current chromosome: 23
  current chromosome: 24
Analyzing: cbs.6
  current chromosome: 1
  current chromosome: 2
  current chromosome: 3
  current chromosome: 4
  current chromosome: 5
  current chromosome: 6
  current chromosome: 7
  current chromosome: 8
```

```
  current chromosome: 9
  current chromosome: 10
  current chromosome: 11
  current chromosome: 12
  current chromosome: 13
  current chromosome: 14
  current chromosome: 15
  current chromosome: 16
  current chromosome: 17
  current chromosome: 18
  current chromosome: 19
  current chromosome: 20
  current chromosome: 21
  current chromosome: 22
  current chromosome: 23
  current chromosome: 24
Analyzing: cbs.7
  current chromosome: 1
  current chromosome: 2
  current chromosome: 3
  current chromosome: 4
  current chromosome: 5
  current chromosome: 6
  current chromosome: 7
  current chromosome: 8
  current chromosome: 9
  current chromosome: 10
  current chromosome: 11
  current chromosome: 12
  current chromosome: 13
  current chromosome: 14
  current chromosome: 15
  current chromosome: 16
  current chromosome: 17
  current chromosome: 18
  current chromosome: 19
  current chromosome: 20
  current chromosome: 21
  current chromosome: 22
  current chromosome: 23
  current chromosome: 24
Analyzing: cbs.8
  current chromosome: 1
  current chromosome: 2
  current chromosome: 3
  current chromosome: 4
  current chromosome: 5
  current chromosome: 6
  current chromosome: 7
  current chromosome: 8
```

```
  current chromosome: 9
  current chromosome: 10
  current chromosome: 11
  current chromosome: 12
  current chromosome: 13
  current chromosome: 14
  current chromosome: 15
  current chromosome: 16
  current chromosome: 17
  current chromosome: 18
  current chromosome: 19
  current chromosome: 20
  current chromosome: 21
  current chromosome: 22
  current chromosome: 23
  current chromosome: 24
Analyzing: cbs.9
  current chromosome: 1
  current chromosome: 2
  current chromosome: 3
  current chromosome: 4
  current chromosome: 5
  current chromosome: 6
  current chromosome: 7
  current chromosome: 8
  current chromosome: 9
  current chromosome: 10
  current chromosome: 11
  current chromosome: 12
  current chromosome: 13
  current chromosome: 14
  current chromosome: 15
  current chromosome: 16
  current chromosome: 17
  current chromosome: 18
  current chromosome: 19
  current chromosome: 20
  current chromosome: 21
  current chromosome: 22
  current chromosome: 23
  current chromosome: 24
Analyzing: cbs.10
  current chromosome: 1
  current chromosome: 2
  current chromosome: 3
  current chromosome: 4
  current chromosome: 5
  current chromosome: 6
  current chromosome: 7
  current chromosome: 8
```

```
  current chromosome: 9
  current chromosome: 10
  current chromosome: 11
  current chromosome: 12
  current chromosome: 13
  current chromosome: 14
  current chromosome: 15
  current chromosome: 16
  current chromosome: 17
  current chromosome: 18
  current chromosome: 19
  current chromosome: 20
  current chromosome: 21
  current chromosome: 22
  current chromosome: 23
  current chromosome: 24
Analyzing: cbs.11
  current chromosome: 1
  current chromosome: 2
  current chromosome: 3
  current chromosome: 4
  current chromosome: 5
  current chromosome: 6
  current chromosome: 7
  current chromosome: 8
  current chromosome: 9
  current chromosome: 10
  current chromosome: 11
  current chromosome: 12
  current chromosome: 13
  current chromosome: 14
  current chromosome: 15
  current chromosome: 16
  current chromosome: 17
  current chromosome: 18
  current chromosome: 19
  current chromosome: 20
  current chromosome: 21
  current chromosome: 22
  current chromosome: 23
  current chromosome: 24
Analyzing: cbs.12
  current chromosome: 1
  current chromosome: 2
  current chromosome: 3
  current chromosome: 4
  current chromosome: 5
  current chromosome: 6
  current chromosome: 7
  current chromosome: 8
```

```
  current chromosome: 9
  current chromosome: 10
  current chromosome: 11
  current chromosome: 12
  current chromosome: 13
  current chromosome: 14
  current chromosome: 15
  current chromosome: 16
  current chromosome: 17
  current chromosome: 18
  current chromosome: 19
  current chromosome: 20
  current chromosome: 21
  current chromosome: 22
  current chromosome: 23
  current chromosome: 24
Analyzing: cbs.13
  current chromosome: 1
  current chromosome: 2
  current chromosome: 3
  current chromosome: 4
  current chromosome: 5
  current chromosome: 6
  current chromosome: 7
  current chromosome: 8
  current chromosome: 9
  current chromosome: 10
  current chromosome: 11
  current chromosome: 12
  current chromosome: 13
  current chromosome: 14
  current chromosome: 15
  current chromosome: 16
  current chromosome: 17
  current chromosome: 18
  current chromosome: 19
  current chromosome: 20
  current chromosome: 21
  current chromosome: 22
  current chromosome: 23
  current chromosome: 24
Analyzing: cbs.14
  current chromosome: 1
  current chromosome: 2
  current chromosome: 3
  current chromosome: 4
  current chromosome: 5
  current chromosome: 6
  current chromosome: 7
  current chromosome: 8
```

```
  current chromosome: 9
  current chromosome: 10
  current chromosome: 11
  current chromosome: 12
  current chromosome: 13
  current chromosome: 14
  current chromosome: 15
  current chromosome: 16
  current chromosome: 17
  current chromosome: 18
  current chromosome: 19
  current chromosome: 20
  current chromosome: 21
  current chromosome: 22
  current chromosome: 23
  current chromosome: 24
Analyzing: cbs.15
  current chromosome: 1
  current chromosome: 2
  current chromosome: 3
  current chromosome: 4
  current chromosome: 5
  current chromosome: 6
  current chromosome: 7
  current chromosome: 8
  current chromosome: 9
  current chromosome: 10
  current chromosome: 11
  current chromosome: 12
  current chromosome: 13
  current chromosome: 14
  current chromosome: 15
  current chromosome: 16
  current chromosome: 17
  current chromosome: 18
  current chromosome: 19
  current chromosome: 20
  current chromosome: 21
  current chromosome: 22
  current chromosome: 23
  current chromosome: 24
Analyzing: cbs.16
  current chromosome: 1
  current chromosome: 2
  current chromosome: 3
  current chromosome: 4
  current chromosome: 5
  current chromosome: 6
  current chromosome: 7
  current chromosome: 8
```

```
  current chromosome: 9
  current chromosome: 10
  current chromosome: 11
  current chromosome: 12
  current chromosome: 13
  current chromosome: 14
  current chromosome: 15
  current chromosome: 16
  current chromosome: 17
  current chromosome: 18
  current chromosome: 19
  current chromosome: 20
  current chromosome: 21
  current chromosome: 22
  current chromosome: 23
  current chromosome: 24
Analyzing: cbs.17
  current chromosome: 1
  current chromosome: 2
  current chromosome: 3
  current chromosome: 4
  current chromosome: 5
  current chromosome: 6
  current chromosome: 7
  current chromosome: 8
  current chromosome: 9
  current chromosome: 10
  current chromosome: 11
  current chromosome: 12
  current chromosome: 13
  current chromosome: 14
  current chromosome: 15
  current chromosome: 16
  current chromosome: 17
  current chromosome: 18
  current chromosome: 19
  current chromosome: 20
  current chromosome: 21
  current chromosome: 22
  current chromosome: 23
  current chromosome: 24
Analyzing: cbs.18
  current chromosome: 1
  current chromosome: 2
  current chromosome: 3
  current chromosome: 4
  current chromosome: 5
  current chromosome: 6
  current chromosome: 7
  current chromosome: 8
```

```
  current chromosome: 9
  current chromosome: 10
  current chromosome: 11
  current chromosome: 12
  current chromosome: 13
  current chromosome: 14
  current chromosome: 15
  current chromosome: 16
  current chromosome: 17
  current chromosome: 18
  current chromosome: 19
  current chromosome: 20
  current chromosome: 21
  current chromosome: 22
  current chromosome: 23
  current chromosome: 24
Analyzing: cbs.19
  current chromosome: 1
  current chromosome: 2
  current chromosome: 3
  current chromosome: 4
  current chromosome: 5
  current chromosome: 6
  current chromosome: 7
  current chromosome: 8
  current chromosome: 9
  current chromosome: 10
  current chromosome: 11
  current chromosome: 12
  current chromosome: 13
  current chromosome: 14
  current chromosome: 15
  current chromosome: 16
  current chromosome: 17
  current chromosome: 18
  current chromosome: 19
  current chromosome: 20
  current chromosome: 21
  current chromosome: 22
  current chromosome: 23
  current chromosome: 24
Analyzing: cbs.20
  current chromosome: 1
  current chromosome: 2
  current chromosome: 3
  current chromosome: 4
  current chromosome: 5
  current chromosome: 6
  current chromosome: 7
  current chromosome: 8
```

```
  current chromosome: 9
  current chromosome: 10
  current chromosome: 11
  current chromosome: 12
  current chromosome: 13
  current chromosome: 14
  current chromosome: 15
  current chromosome: 16
  current chromosome: 17
  current chromosome: 18
  current chromosome: 19
  current chromosome: 20
  current chromosome: 21
  current chromosome: 22
  current chromosome: 23
  current chromosome: 24
Analyzing: cbs.21
  current chromosome: 1
  current chromosome: 2
  current chromosome: 3
  current chromosome: 4
  current chromosome: 5
  current chromosome: 6
  current chromosome: 7
  current chromosome: 8
  current chromosome: 9
  current chromosome: 10
  current chromosome: 11
  current chromosome: 12
  current chromosome: 13
  current chromosome: 14
  current chromosome: 15
  current chromosome: 16
  current chromosome: 17
  current chromosome: 18
  current chromosome: 19
  current chromosome: 20
  current chromosome: 21
  current chromosome: 22
  current chromosome: 23
  current chromosome: 24
Analyzing: cbs.22
  current chromosome: 1
  current chromosome: 2
  current chromosome: 3
  current chromosome: 4
  current chromosome: 5
  current chromosome: 6
  current chromosome: 7
  current chromosome: 8
```

```
  current chromosome: 9
  current chromosome: 10
  current chromosome: 11
  current chromosome: 12
  current chromosome: 13
  current chromosome: 14
  current chromosome: 15
  current chromosome: 16
  current chromosome: 17
  current chromosome: 18
  current chromosome: 19
  current chromosome: 20
  current chromosome: 21
  current chromosome: 22
  current chromosome: 23
  current chromosome: 24
Analyzing: cbs.23
  current chromosome: 1
  current chromosome: 2
  current chromosome: 3
  current chromosome: 4
  current chromosome: 5
  current chromosome: 6
  current chromosome: 7
  current chromosome: 8
  current chromosome: 9
  current chromosome: 10
  current chromosome: 11
  current chromosome: 12
  current chromosome: 13
  current chromosome: 14
  current chromosome: 15
  current chromosome: 16
  current chromosome: 17
  current chromosome: 18
  current chromosome: 19
  current chromosome: 20
  current chromosome: 21
  current chromosome: 22
  current chromosome: 23
  current chromosome: 24
Analyzing: cbs.24
  current chromosome: 1
  current chromosome: 2
  current chromosome: 3
  current chromosome: 4
  current chromosome: 5
  current chromosome: 6
  current chromosome: 7
  current chromosome: 8
```

```
  current chromosome: 9
  current chromosome: 10
  current chromosome: 11
  current chromosome: 12
  current chromosome: 13
  current chromosome: 14
  current chromosome: 15
  current chromosome: 16
  current chromosome: 17
  current chromosome: 18
  current chromosome: 19
  current chromosome: 20
  current chromosome: 21
  current chromosome: 22
  current chromosome: 23
  current chromosome: 24
Analyzing: cbs.25
  current chromosome: 1
  current chromosome: 2
  current chromosome: 3
  current chromosome: 4
  current chromosome: 5
  current chromosome: 6
  current chromosome: 7
  current chromosome: 8
  current chromosome: 9
  current chromosome: 10
  current chromosome: 11
  current chromosome: 12
  current chromosome: 13
  current chromosome: 14
  current chromosome: 15
  current chromosome: 16
  current chromosome: 17
  current chromosome: 18
  current chromosome: 19
  current chromosome: 20
  current chromosome: 21
  current chromosome: 22
  current chromosome: 23
  current chromosome: 24
Done
```

```
> basal25cbs <- callCGHStatesThreshold(basal25cbs, gainthresh = 0.08, ampthresh = 0.45,
+     contig = 3)
```

```
thresholding aCGH data
1         2         3         4         5         6         7         8         9         10         11
1         2         3         4         5         6         7         8         9         10         11
1         2         3         4         5         6         7         8         9         10         11
```

```
1       2       3       4       5       6       7       8       9       10      11
1       2       3       4       5       6       7       8       9       10      11
1       2       3       4       5       6       7       8       9       10      11
1       2       3       4       5       6       7       8       9       10      11
1       2       3       4       5       6       7       8       9       10      11
1       2       3       4       5       6       7       8       9       10      11
1       2       3       4       5       6       7       8       9       10      11
1       2       3       4       5       6       7       8       9       10      11
1       2       3       4       5       6       7       8       9       10      11
1       2       3       4       5       6       7       8       9       10      11
1       2       3       4       5       6       7       8       9       10      11
1       2       3       4       5       6       7       8       9       10      11
1       2       3       4       5       6       7       8       9       10      11
1       2       3       4       5       6       7       8       9       10      11
1       2       3       4       5       6       7       8       9       10      11
1       2       3       4       5       6       7       8       9       10      11
1       2       3       4       5       6       7       8       9       10      11
1       2       3       4       5       6       7       8       9       10      11
1       2       3       4       5       6       7       8       9       10      11
1       2       3       4       5       6       7       8       9       10      11
1       2       3       4       5       6       7       8       9       10      11
1       2       3       4       5       6       7       8       9       10      11
Filtering contiguous states...Done
Imputing missing values
1       2       3       4       5       6       7       8       9       10      11
1       2       3       4       5       6       7       8       9       10      11
1       2       3       4       5       6       7       8       9       10      11
1       2       3       4       5       6       7       8       9       10      11
1       2       3       4       5       6       7       8       9       10      11
1       2       3       4       5       6       7       8       9       10      11
1       2       3       4       5       6       7       8       9       10      11
1       2       3       4       5       6       7       8       9       10      11
1       2       3       4       5       6       7       8       9       10      11
1       2       3       4       5       6       7       8       9       10      11
1       2       3       4       5       6       7       8       9       10      11
1       2       3       4       5       6       7       8       9       10      11
1       2       3       4       5       6       7       8       9       10      11
1       2       3       4       5       6       7       8       9       10      11
1       2       3       4       5       6       7       8       9       10      11
1       2       3       4       5       6       7       8       9       10      11
1       2       3       4       5       6       7       8       9       10      11
1       2       3       4       5       6       7       8       9       10      11
1       2       3       4       5       6       7       8       9       10      11
1       2       3       4       5       6       7       8       9       10      11
1       2       3       4       5       6       7       8       9       10      11
1       2       3       4       5       6       7       8       9       10      11
1       2       3       4       5       6       7       8       9       10      11
1       2       3       4       5       6       7       8       9       10      11
1       2       3       4       5       6       7       8       9       10      11
Done
```

```
> validObject(basal25cbs)

[1] TRUE

> spitTables(basal25cbs, "BCCL25.aCGH")

writing featureData table
Writing phenoData table
Writing A table
Writing exprs table
Writing flags table
Writing GL table
Writing smo table
Writing GLAD table
Done
```

Illustrate genomic aberration with frequency plots

```
> plotFrequency(basal25cbs, project = "BCCL25", device = "PDF")

pdf
  2

> basal25cbs <- readPheno(basal25cbs, paste(filePathStartingFiles, "Basal.25.phenoUpdated20110104.txt",
+     sep = ""))

Reading pheno file
25 Pheno sampleNames
25 Eset sampleNames
25 Matching sampleNames
 BT20 BT474 BT483 BT549 HCC1143 HCC1187 HCC1428 HCC1569 HCC1937 HCC1954 HCC38 HCC70 HS578T MDAMB157 MDAMI
Done

> plotFrequencyLattice(basal25cbs, basal25cbs$expression.cluster, project = "BCCL25.3ExpressionClusters",
+     device = "PDF")

pdf
  2
```

Compare the genomic aberration between the different BCCL groups and illustrate them with frequency plots

```
> basal25cbs.FE <- fisherTestCGH(basal25cbs, basal25cbs$expression.cluster, project = "BCCL25.3Expressio

Performing Fishers Exact tests for gains, losses, amps and dels in BasalA BasalB and Luminal groups
100  200  300  400  500  600  700  800  900  1000  1100  1200  1300  1400  1500  1600  1700  1800  1900
Done

> listBreaksFisher(basal25cbs.FE, project = "BCCL25.3ExpressionClusters")
> latticePlotFishers(basal25cbs.FE, project = "BCCL25.3ExpressionClusters", device = "PDF")

pdf
  2
```

Record the genomic aberrations based on the thresholds gainthresh=0.08, ampthresh=0.45 for each sample individually, saved in folder sample.GALS

```
> listBreaksGL(basal25cbs, contig = 3, project = "BCCL25")
```

```
BT20
BT474
BT483
BT549
HCC1143
HCC1187
HCC1428
HCC1569
HCC1937
HCC1954
HCC38
HCC70
HS578T
MDAMB157
MDAMB231
MDAMB436
MDAMB468
SKBR3
SUM1315
SUM149
SUM159
SUM190
SUM225
T47D
ZR7530
```

Record the genomic aberrations based on the thresholds gainthresh=0.08, ampthresh=0.45 occurring in at least 4 samples for gains and at least 3 samples for amplifications

```
> listBreaksGLAD(basal25cbs, contig = 3, gain.count = 4, amp.count = 1, project = "BCCL25")
```

Determine the genomic instability (GI) for each BCCL and compare the GI between the different BCCL groups

```
> basal25cbs <- countEventsCGH(basal25cbs, project = "BCCL25", return.CGH = T)
> range(basal25cbs$total.prop)
```

```
[1] 0.137 0.838
```

```
> range(basal25cbs$amp.prop)
```

```
[1] 0.00127 0.05790
```

```
> range(basal25cbs$del.prop)
```

```
[1] 0.00098 0.02170
```

```
> summary(lm(basal25cbs$amps ~ basal25cbs$expression.cluster))

Call:
lm(formula = basal25cbs$amps ~ basal25cbs$expression.cluster)

Residuals:
   Min     1Q Median     3Q    Max 
-19.60  -7.60   0.25   5.40  14.86 

Coefficients:
                                     Estimate Std. Error t value Pr(>|t|)    
(Intercept)                            26.600      3.245   8.197 3.93e-08 ***
basal25cbs$expression.clusterBasalB   -11.457      5.057  -2.266   0.0337 *  
basal25cbs$expression.clusterLuminal    1.150      4.868   0.236   0.8154    
---
Signif. codes:  0 âĂŸ***âĂŹ 0.001 âĂŸ**âĂŹ 0.01 âĂŸ*âĂŹ 0.05 âĂŸ.âĂŹ 0.1 âĂŸ âĂŹ 1 

Residual standard error: 10.26 on 22 degrees of freedom
Multiple R-squared: 0.2391,	Adjusted R-squared: 0.1699 
F-statistic: 3.456 on 2 and 22 DF,  p-value: 0.04954 
```

Illustrate the distribution of GI in the different BCCL groups with boxplots and test their significant difference

```
> pdf("BCCL25.genomicInstability.pdf")
> par(mfrow = c(3, 1))
> boxplot(basal25cbs$total.counts ~ basal25cbs$expression.cluster, main = "Total aberrations in BCCL")
> boxplot(basal25cbs$amps ~ basal25cbs$expression.cluster, main = "Amplifications in BCCL")
> boxplot(basal25cbs$dels ~ basal25cbs$expression.cluster, main = "Deletion in BCCL")
> dev.off()

pdf 
  2 

> t.test(basal25cbs$total.counts[basal25cbs$expression.cluster == "BasalA"], basal25cbs$total.counts[bas
+     "BasalB"])

	Welch Two Sample t-test

data:  basal25cbs$total.counts[basal25cbs$expression.cluster == "BasalA"] and basal25cbs$total.counts[bas
t = 1.8949, df = 12.059, p-value = 0.08234
alternative hypothesis: true difference in means is not equal to 0 
95 percent confidence interval:
  -7.118604 102.518604 
sample estimates:
mean of x mean of y 
    326.7     279.0 

> t.test(basal25cbs$total.counts[basal25cbs$expression.cluster == "BasalA"], basal25cbs$total.counts[bas
+     "Luminal"])
```

```
        Welch Two Sample t-test

data:  basal25cbs$total.counts[basal25cbs$expression.cluster == "BasalA"] and basal25cbs$total.counts[bas
t = -0.2475, df = 15.993, p-value = 0.8077
alternative hypothesis: true difference in means is not equal to 0
95 percent confidence interval:
 -47.1175  37.2675
sample estimates:
mean of x mean of y
  326.700   331.625

> t.test(basal25cbs$total.counts[basal25cbs$expression.cluster == "BasalB"], basal25cbs$total.counts[basa
+     "Luminal"])

        Welch Two Sample t-test

data:  basal25cbs$total.counts[basal25cbs$expression.cluster == "BasalB"] and basal25cbs$total.counts[bas
t = -2.1927, df = 10.476, p-value = 0.05191
alternative hypothesis: true difference in means is not equal to 0
95 percent confidence interval:
 -105.7738516    0.5238516
sample estimates:
mean of x mean of y
  279.000   331.625

> t.test(basal25cbs$amps[basal25cbs$expression.cluster == "BasalA"], basal25cbs$amps[basal25cbs$expressio
+     "BasalB"])

        Welch Two Sample t-test

data:  basal25cbs$amps[basal25cbs$expression.cluster == "BasalA"] and basal25cbs$amps[basal25cbs$expressi
t = 2.2723, df = 12.819, p-value = 0.04096
alternative hypothesis: true difference in means is not equal to 0
95 percent confidence interval:
  0.5488997 22.3653860
sample estimates:
mean of x mean of y
 26.60000  15.14286

> t.test(basal25cbs$amps[basal25cbs$expression.cluster == "BasalA"], basal25cbs$amps[basal25cbs$expressio
+     "Luminal"])

        Welch Two Sample t-test

data:  basal25cbs$amps[basal25cbs$expression.cluster == "BasalA"] and basal25cbs$amps[basal25cbs$expressi
t = -0.236, df = 14.873, p-value = 0.8167
alternative hypothesis: true difference in means is not equal to 0
95 percent confidence interval:
 -11.545033   9.245033
sample estimates:
mean of x mean of y
    26.60     27.75
```

```
> t.test(basal25cbs$amps[basal25cbs$expression.cluster == "BasalB"], basal25cbs$amps[basal25cbs$expressio
+     "Luminal"])

        Welch Two Sample t-test

data:  basal25cbs$amps[basal25cbs$expression.cluster == "BasalB"] and basal25cbs$amps[basal25cbs$expressi
t = -2.3435, df = 12.767, p-value = 0.03598
alternative hypothesis: true difference in means is not equal to 0
95 percent confidence interval:
 -24.2506544  -0.9636314
sample estimates:
mean of x mean of y
 15.14286  27.75000

> t.test(basal25cbs$dels[basal25cbs$expression.cluster == "BasalA"], basal25cbs$dels[basal25cbs$expressio
+     "BasalB"])

        Welch Two Sample t-test

data:  basal25cbs$dels[basal25cbs$expression.cluster == "BasalA"] and basal25cbs$dels[basal25cbs$expressi
t = 2.0097, df = 14.935, p-value = 0.06289
alternative hypothesis: true difference in means is not equal to 0
95 percent confidence interval:
 -0.4207635 14.2207635
sample estimates:
mean of x mean of y
     14.9       8.0

> t.test(basal25cbs$dels[basal25cbs$expression.cluster == "Luminal"], basal25cbs$dels[basal25cbs$expressi
+     "BasalB"])

        Welch Two Sample t-test

data:  basal25cbs$dels[basal25cbs$expression.cluster == "Luminal"] and basal25cbs$dels[basal25cbs$express
t = 2.7529, df = 12.427, p-value = 0.01703
alternative hypothesis: true difference in means is not equal to 0
95 percent confidence interval:
  2.036197 17.213803
sample estimates:
mean of x mean of y
   17.625     8.000

> t.test(basal25cbs$dels[basal25cbs$expression.cluster == "Luminal"], basal25cbs$dels[basal25cbs$expressi
+     "BasalA"])

        Welch Two Sample t-test

data:  basal25cbs$dels[basal25cbs$expression.cluster == "Luminal"] and basal25cbs$dels[basal25cbs$express
t = 0.6956, df = 15.626, p-value = 0.4969
alternative hypothesis: true difference in means is not equal to 0
95 percent confidence interval:
```

```
 -5.595427 11.045427
sample estimates:
mean of x mean of y
   17.625    14.900
```

Split the genomic data according the BCCL expression clusters

```
> basal25cbs.Luminal <- basal25cbs[, basal25cbs$expression.cluster == "Luminal"]
> basal25cbs.BasalA <- basal25cbs[, basal25cbs$expression.cluster == "BasalA"]
> basal25cbs.BasalB <- basal25cbs[, basal25cbs$expression.cluster == "BasalB"]
```

Plot the genomic aberration according the BCCL expression clusters

```
> plotFrequency(basal25cbs.BasalA, project = "basal25cbs.BasalA", device = "PDF")

pdf
  2

> plotFrequency(basal25cbs.BasalB, project = "basal25cbs.BasalB", device = "PDF")

pdf
  2
```

Record the changes specific for each expression clusters and plot the specific aberration (Figure 3 in manuscript)

```
> listBreaksGLAD(basal25cbs.Luminal, contig = 3, gain.count = 2, amp.count = 1,
+     project = "BCCL.Luminal")
> listBreaksGLAD(basal25cbs.BasalA, contig = 3, gain.count = 2, amp.count = 1,
+     project = "BCCL.BasalA")
> listBreaksGLAD(basal25cbs.BasalB, contig = 3, gain.count = 2, amp.count = 1,
+     project = "BCCL.BasalB")
> basal25cbs.Basal <- basal25cbs[, basal25cbs$expression.cluster != "Luminal"]
> basal.group <- c("BasalA", "BasalA", "BasalA", "BasalA", "BasalA", "BasalA",
+     "BasalA", "BasalA", "BasalA", "BasalA", "BasalB", "BasalB", "BasalB", "BasalB",
+     "BasalB", "BasalB", "BasalB")
> pData(basal25cbs.Basal)$basal.group <- basal.group
> basal25cbs.Basal.FE <- fisherTestCGH(basal25cbs.Basal, basal25cbs.Basal$basal.group,
+     project = "BCCL25.Basal")

Performing Fishers Exact tests for gains, losses, amps and dels in BasalA and BasalB groups
100  200  300  400  500  600  700  800  900  1000  1100  1200  1300  1400  1500  1600  1700  1800  1900
Done

> listBreaksFisher(basal25cbs.Basal.FE, project = "BCCL25.Basal")
> latticePlotFishers(basal25cbs.Basal.FE, project = "BCCL25.Basal", device = "PDF")

pdf
  2

> basal25cbs.Basal.FE2 <- fisherTestCGH(basal25cbs.Basal, basal25cbs.Basal$basal.group,
+     p.val.adjustment = NULL, project = "BCCL25.Basal.NoPadjust2")
```

```
Performing Fishers Exact tests for gains, losses, amps and dels in BasalA and BasalB groups
100  200  300  400  500  600  700  800  900  1000  1100  1200  1300  1400  1500  1600  1700  1800  1900
Done
```

```
> listBreaksFisher(basal25cbs.Basal.FE2, project = "BCCL25.BasalNoPadjust")
> latticePlotFishers(basal25cbs.Basal.FE2, project = "BCCL25.BasalNoPadjust",
+     plot.adjusted.p = FALSE, device = "PDF")
```

```
pdf
  2
```

```
> setwd("..")
```

Compare the BCCL with data from 56 TNBC Turner et al., Oncogene 2010, so read in the TNT aCGH from tables

```
> setwd(paste(filePath, "BCCL.genomicAnalysis/TNT56.aCGH/", sep = ""))
> TNT <- readBACE.cgh(exprsFile = "TNT56.exprs.txt", smoDataFile = "TNT56.CBS.txt",
+     featureDataFile = "TNT.56.fdata.txt", stringsAsFactors = F, row.names = 1)
> validObject(TNT)
```

```
[1] TRUE
```

```
> TNT <- callCGHStatesThreshold(TNT, gainthresh = 0.08, ampthresh = 0.45, contig = 3)
```

```
thresholding aCGH data
1    2    3    4    5    6    7    8    9    10    11
1    2    3    4    5    6    7    8    9    10    11
1    2    3    4    5    6    7    8    9    10    11
1    2    3    4    5    6    7    8    9    10    11
1    2    3    4    5    6    7    8    9    10    11
1    2    3    4    5    6    7    8    9    10    11
1    2    3    4    5    6    7    8    9    10    11
1    2    3    4    5    6    7    8    9    10    11
1    2    3    4    5    6    7    8    9    10    11
1    2    3    4    5    6    7    8    9    10    11
1    2    3    4    5    6    7    8    9    10    11
1    2    3    4    5    6    7    8    9    10    11
1    2    3    4    5    6    7    8    9    10    11
1    2    3    4    5    6    7    8    9    10    11
1    2    3    4    5    6    7    8    9    10    11
1    2    3    4    5    6    7    8    9    10    11
1    2    3    4    5    6    7    8    9    10    11
1    2    3    4    5    6    7    8    9    10    11
1    2    3    4    5    6    7    8    9    10    11
1    2    3    4    5    6    7    8    9    10    11
1    2    3    4    5    6    7    8    9    10    11
1    2    3    4    5    6    7    8    9    10    11
1    2    3    4    5    6    7    8    9    10    11
1    2    3    4    5    6    7    8    9    10    11
1    2    3    4    5    6    7    8    9    10    11
1    2    3    4    5    6    7    8    9    10    11
```

```
1         2         3         4         5         6         7         8         9         10        11
1         2         3         4         5         6         7         8         9         10        11
1         2         3         4         5         6         7         8         9         10        11
1         2         3         4         5         6         7         8         9         10        11
1         2         3         4         5         6         7         8         9         10        11
1         2         3         4         5         6         7         8         9         10        11
1         2         3         4         5         6         7         8         9         10        11
1         2         3         4         5         6         7         8         9         10        11
1         2         3         4         5         6         7         8         9         10        11
1         2         3         4         5         6         7         8         9         10        11
1         2         3         4         5         6         7         8         9         10        11
1         2         3         4         5         6         7         8         9         10        11
1         2         3         4         5         6         7         8         9         10        11
1         2         3         4         5         6         7         8         9         10        11
1         2         3         4         5         6         7         8         9         10        11
1         2         3         4         5         6         7         8         9         10        11
1         2         3         4         5         6         7         8         9         10        11
1         2         3         4         5         6         7         8         9         10        11
1         2         3         4         5         6         7         8         9         10        11
1         2         3         4         5         6         7         8         9         10        11
1         2         3         4         5         6         7         8         9         10        11
1         2         3         4         5         6         7         8         9         10        11
1         2         3         4         5         6         7         8         9         10        11
1         2         3         4         5         6         7         8         9         10        11
1         2         3         4         5         6         7         8         9         10        11
1         2         3         4         5         6         7         8         9         10        11
1         2         3         4         5         6         7         8         9         10        11
1         2         3         4         5         6         7         8         9         10        11
1         2         3         4         5         6         7         8         9         10        11
1         2         3         4         5         6         7         8         9         10        11
Filtering contiguous states...Done
Imputing missing values
1         2         3         4         5         6         7         8         9         10        11
1         2         3         4         5         6         7         8         9         10        11
1         2         3         4         5         6         7         8         9         10        11
1         2         3         4         5         6         7         8         9         10        11
1         2         3         4         5         6         7         8         9         10        11
1         2         3         4         5         6         7         8         9         10        11
1         2         3         4         5         6         7         8         9         10        11
1         2         3         4         5         6         7         8         9         10        11
1         2         3         4         5         6         7         8         9         10        11
1         2         3         4         5         6         7         8         9         10        11
1         2         3         4         5         6         7         8         9         10        11
1         2         3         4         5         6         7         8         9         10        11
1         2         3         4         5         6         7         8         9         10        11
1         2         3         4         5         6         7         8         9         10        11
1         2         3         4         5         6         7         8         9         10        11
1         2         3         4         5         6         7         8         9         10        11
1         2         3         4         5         6         7         8         9         10        11
1         2         3         4         5         6         7         8         9         10        11
```

```
1    2    3    4    5    6    7    8    9    10   11
1    2    3    4    5    6    7    8    9    10   11
1    2    3    4    5    6    7    8    9    10   11
1    2    3    4    5    6    7    8    9    10   11
1    2    3    4    5    6    7    8    9    10   11
1    2    3    4    5    6    7    8    9    10   11
1    2    3    4    5    6    7    8    9    10   11
1    2    3    4    5    6    7    8    9    10   11
1    2    3    4    5    6    7    8    9    10   11
1    2    3    4    5    6    7    8    9    10   11
1    2    3    4    5    6    7    8    9    10   11
1    2    3    4    5    6    7    8    9    10   11
1    2    3    4    5    6    7    8    9    10   11
1    2    3    4    5    6    7    8    9    10   11
1    2    3    4    5    6    7    8    9    10   11
1    2    3    4    5    6    7    8    9    10   11
1    2    3    4    5    6    7    8    9    10   11
1    2    3    4    5    6    7    8    9    10   11
1    2    3    4    5    6    7    8    9    10   11
1    2    3    4    5    6    7    8    9    10   11
1    2    3    4    5    6    7    8    9    10   11
1    2    3    4    5    6    7    8    9    10   11
1    2    3    4    5    6    7    8    9    10   11
1    2    3    4    5    6    7    8    9    10   11
1    2    3    4    5    6    7    8    9    10   11
1    2    3    4    5    6    7    8    9    10   11
1    2    3    4    5    6    7    8    9    10   11
1    2    3    4    5    6    7    8    9    10   11
1    2    3    4    5    6    7    8    9    10   11
1    2    3    4    5    6    7    8    9    10   11
1    2    3    4    5    6    7    8    9    10   11
1    2    3    4    5    6    7    8    9    10   11
1    2    3    4    5    6    7    8    9    10   11
1    2    3    4    5    6    7    8    9    10   11
1    2    3    4    5    6    7    8    9    10   11
1    2    3    4    5    6    7    8    9    10   11
1    2    3    4    5    6    7    8    9    10   11
1    2    3    4    5    6    7    8    9    10   11
Done
```

```
> plotFrequency(TNT, project = "TNT56", device = "PDF")
```

```
pdf
  2
```

```
> listBreaksGLAD(TNT, contig = 3, gain.count = 3, amp.count = 2, project = "TNT56")
> TNT <- countEventsCGH(TNT, project = "TNT56", return.CGH = T)
```

Reorder the basal25cbs to be in alignment with expression data and plot the genomic aberrations as shingle plots and heatmaps

```
> basal25cbs <- basal25cbs[, order(basal25cbs$cluster.order, decreasing = T)]
> frequencyShingles(TNT, main = "Triple Negative Breast Cancers", project = "TNT56",
+     device = "PDF")

Listing aCGH states
1         2         3         4         5         6         7         8         9         10        11
  2

> frequencyShingles(basal25cbs, main = "Breast Cancer Cell Lines", project = "BCCL25",
+     device = "PDF")

Listing aCGH states
1         2         3         4         5         6         7         8         9         10        11
  2

> frequencyShingles(basal25cbs.Basal, main = "Breast Cancer Cell Lines", project = "BCCL25.Basal",
+     device = "PDF")

Listing aCGH states
1         2         3         4         5         6         7         8         9         10        11
  2

> cghHeatmap(TNT, project = "TNT56", device = "PNG", main = "Triple Negative Breast Cancers")

pdf
  2

> cghHeatmap(basal25cbs, cluster = NULL, project = "BCCL25.expression.clustered",
+     device = "PNG", main = "Breast Cancer Cell Lines")

pdf
  2

> cghHeatmap(basal25cbs, project = "BCCL25.aCGH.clustered", device = "PNG", main = "Breast Cancer Cell Li

pdf
  2
```

Investigate the genomic aberration on the gene level for each expression cluster separately

```
> all.genes.table <- read.delim(paste(filePathStartingFiles, "all.genes.txt",
+     sep = ""), sep = "\t", stringsAsFactors = F, header = T)
> TNT.amplicons <- read.table("TNT.recurrent.amplicons.txt", header = T, sep = "\t",
+     stringsAsFactors = F)
> gains <- losses <- amps <- dels <- gain.cases <- loss.cases <- amp.cases <- del.cases <- rep(NA,
+     nrow(TNT.amplicons))
> cgh <- basal25cbs
> for (i in 1:nrow(TNT.amplicons)) {
+     iprobes.cgh.chrom <- fData(cgh)[which(fData(cgh)$chrom == TNT.amplicons$chrom[i]),
+         , drop = F]
+     iprobes.cgh.plus <- iprobes.cgh.chrom[which(iprobes.cgh.chrom$end >= TNT.amplicons$start[i]),
+         , drop = F]
```

```
+     iprobes.gene.Minus <- iprobes.cgh.chrom[which(iprobes.cgh.chrom$start <=
+         TNT.amplicons$end[i]), , drop = F]
+     iprobes.cgh.overlap <- fData(cgh)[which(is.element(featureNames(cgh), intersect(row.names(iprobes.
+         row.names(iprobes.gene.Minus)))), , drop = F]
+     iprobes.gene.More <- iprobes.cgh.chrom[which(iprobes.cgh.chrom$start > all.genes.table$start[i]),
+         , drop = F]
+     iprobes.cgh.less <- iprobes.cgh.chrom[which(iprobes.cgh.chrom$end < all.genes.table$end[i]),
+         , drop = F]
+     iprobes.gene.More <- iprobes.gene.More[order(iprobes.gene.More$start), ,
+         drop = F]
+     iprobes.cgh.less <- iprobes.cgh.less[rev(order(iprobes.cgh.less$start)),
+         , drop = F]
+     if (nrow(iprobes.cgh.overlap) < 1) {
+         iprobes.cgh <- rbind(iprobes.cgh.less[1, ], iprobes.gene.More[1, ])
+         iprobes.cgh <- iprobes.cgh[order(iprobes.cgh$start), ]
+     }
+     else {
+         iprobes.cgh <- iprobes.cgh.overlap[order(iprobes.cgh.overlap$start),
+             ]
+     }
+     iGL <- as.data.frame(assayData(cgh)$GL[which(is.element(featureNames(cgh),
+         row.names(iprobes.cgh))), , drop = F])
+     gains[i] <- sum(apply(iGL == 1, 2, max) == 1)
+     gain.cases[i] <- paste(sampleNames(cgh)[which(apply(iGL == 1, 2, max) ==
+         1)], collapse = ", ")
+     losses[i] <- sum(apply(iGL == -1, 2, max) == 1)
+     loss.cases[i] <- paste(sampleNames(cgh)[which(apply(iGL == -1, 2, max) ==
+         1)], collapse = ", ")
+     amps[i] <- sum(apply(iGL == 2, 2, max) == 1)
+     amp.cases[i] <- paste(sampleNames(cgh)[which(apply(iGL == 2, 2, max) ==
+         1)], collapse = ", ")
+     dels[i] <- sum(apply(iGL == -2, 2, max) == 1)
+     del.cases[i] <- paste(sampleNames(cgh)[which(apply(iGL == -2, 2, max) ==
+         1)], collapse = ", ")
+     rm(iprobes.cgh)
+ }
> TNT.total.amplicons <- data.frame(TNT.amplicons, gains, gain.cases, losses,
+     loss.cases, amps, amp.cases, dels, del.cases)
> write.table(TNT.total.amplicons, "TNT.amplicons.all.lines.xls", row.names = F,
+     sep = "\t", na = "")
> setwd("..")
```

Compress the genomic data on the gene level

```
> dir.create("GeneLevel_GenomicAnalysis")
> setwd("GeneLevel_GenomicAnalysis")
> basal25cbs.geneCGH <- geneCGH(basal25cbs, genes.table = paste(filePathStartingFiles,
+     "all.genes.txt", sep = ""), project = "BCCL25")
```

```
Listing aCGH states for 18522 genes
100  200  300  400  500  600  700  800  900  1000  1100  1200  1300  1400  1500  1600  1700  1800  1900
```

```
Done
```

```
> TNT.geneCGH <- geneCGH(TNT, genes.table = paste(filePathStartingFiles, "all.genes.txt",
+     sep = ""), project = "TNT56")
```

```
Listing aCGH states for 18522 genes
100  200  300  400  500  600  700  800  900  1000  1100  1200  1300  1400  1500  1600  1700  1800  1900
Done
```

Create a combined gene expression and genomic expression set and determine the correlation between expression and copy-number levels

```
> basal25expcgh <- medianCGH(Basal.25.assembly55.lumi.mapped, basal25cbs)
```

```
Matching expression and aCGH sampleNames
Expression sampleNames
HCC1937 HCC70 MDAMB468 SUM159 HCC1428 SUM149 HCC1954 MDAMB231 BT483 MDAMB157 HS578T MDAMB436 HCC1143 HCC3
aCGH sampleNames
HCC1569 HCC1187 HCC38 HCC1143 HCC1937 SUM149 BT20 HCC1954 MDAMB468 HCC70 MDAMB231 SUM159 MDAMB436 SUM1315
25 matching sampleNames
HCC1937 HCC70 MDAMB468 SUM159 HCC1428 SUM149 HCC1954 MDAMB231 BT483 MDAMB157 HS578T MDAMB436 HCC1143 HCC3
Calculating median CGH values for expression probes
100  200  300  400  500  600  700  800  900  1000  1100  1200  1300  1400  1500  1600  1700  1800  1900
1         2         3         4         5         6         7         8         9         10         11
1         2         3         4         5         6         7         8         9         10         11
1         2         3         4         5         6         7         8         9         10         11
1         2         3         4         5         6         7         8         9         10         11
1         2         3         4         5         6         7         8         9         10         11
1         2         3         4         5         6         7         8         9         10         11
1         2         3         4         5         6         7         8         9         10         11
1         2         3         4         5         6         7         8         9         10         11
1         2         3         4         5         6         7         8         9         10         11
1         2         3         4         5         6         7         8         9         10         11
1         2         3         4         5         6         7         8         9         10         11
1         2         3         4         5         6         7         8         9         10         11
1         2         3         4         5         6         7         8         9         10         11
1         2         3         4         5         6         7         8         9         10         11
1         2         3         4         5         6         7         8         9         10         11
1         2         3         4         5         6         7         8         9         10         11
1         2         3         4         5         6         7         8         9         10         11
1         2         3         4         5         6         7         8         9         10         11
1         2         3         4         5         6         7         8         9         10         11
1         2         3         4         5         6         7         8         9         10         11
1         2         3         4         5         6         7         8         9         10         11
1         2         3         4         5         6         7         8         9         10         11
1         2         3         4         5         6         7         8         9         10         11
1         2         3         4         5         6         7         8         9         10         11
Imputing missing values
1         2         3         4         5         6         7         8         9         10         11
1         2         3         4         5         6         7         8         9         10         11
1         2         3         4         5         6         7         8         9         10         11
```

```
1         2         3         4         5         6         7         8         9         10         11
1         2         3         4         5         6         7         8         9         10         11
1         2         3         4         5         6         7         8         9         10         11
1         2         3         4         5         6         7         8         9         10         11
1         2         3         4         5         6         7         8         9         10         11
1         2         3         4         5         6         7         8         9         10         11
1         2         3         4         5         6         7         8         9         10         11
1         2         3         4         5         6         7         8         9         10         11
1         2         3         4         5         6         7         8         9         10         11
1         2         3         4         5         6         7         8         9         10         11
1         2         3         4         5         6         7         8         9         10         11
1         2         3         4         5         6         7         8         9         10         11
1         2         3         4         5         6         7         8         9         10         11
1         2         3         4         5         6         7         8         9         10         11
1         2         3         4         5         6         7         8         9         10         11
1         2         3         4         5         6         7         8         9         10         11
1         2         3         4         5         6         7         8         9         10         11
1         2         3         4         5         6         7         8         9         10         11
1         2         3         4         5         6         7         8         9         10         11
1         2         3         4         5         6         7         8         9         10         11
1         2         3         4         5         6         7         8         9         10         11
1         2         3         4         5         6         7         8         9         10         11
Done

> basal25expcgh <- expressionCGHCorrelation(basal25expcgh)

Correlation of gene expression with aCGH
100  200  300  400  500  600  700  800  900  1000  1100  1200  1300  1400  1500  1600  1700  1800  1900
Done
6763 significantly correlated genes

Done

> length(unique(fData(basal25expcgh)$ensg)[fData(basal25expcgh)$pearson.adjp <
+     0.05])

[1] 6763

> length(unique(fData(basal25expcgh)$symbol[fData(basal25expcgh)$pearson.adjp <
+     0.05]))

[1] 5834

> basal25expcgh.uSymbol <- removeReplicateProbesByCorrelation(basal25expcgh, eset.cor = "pearson.adjp",
+     eset.id = "symbol", verbose = T)

 18393 probes remaining
 Done

> length(unique(fData(basal25expcgh.uSymbol)$symbol[fData(basal25expcgh.uSymbol)$pearson.adjp <
+     0.05]))
```

```
[1] 4558

> spitTables(basal25expcgh.uSymbol, "basal25expcgh.uSymbol")

writing featureData table
Writing phenoData table
Writing beadNum table
Writing cgh.exprs table
Writing detection table
Writing exprs table
Writing flags table
Writing GL table
Writing se.exprs table
Writing smo table
Writing GLAD table
Done

> pdf("DistributionOfCNdependentGEX.pdf")
> barplot(table(fData(basal25expcgh)$chrom[fData(basal25expcgh)$pearson.adjp <
+     0.05]), xlab = "Chromosome", main = "Distribution of CN-dependent GEX",
+     ylab = "Number of genes")
> dev.off()

pdf
  2

> basal25expcgh <- wilcoxTestCGH(basal25expcgh)

Wilcoxon rank sum tests of expression based on copy number changes
100  200  300  400  500  600  700  800  900  1000  1100  1200  1300  1400  1500  1600  1700  1800  1900
Done
664 genes overexpressed when amplified

Done

> length(unique(fData(basal25expcgh)$ensg[fData(basal25expcgh)$Wilcox.adjp.amp <
+     0.05]))

[1] 278

> length(unique(fData(basal25expcgh)$ensg[fData(basal25expcgh)$Wilcox.adjp.del <
+     0.05]))

[1] 52

> length(unique(fData(basal25expcgh.uSymbol)$ensg[fData(basal25expcgh.uSymbol)$Wilcox.adjp.del <
+     0.05]))

[1] 0
```

Graphical illustration how an area of copy number changes influences gene expression (Splitheatmap for Figure 4)

```
> splitHeatmap(basal25expcgh.uSymbol, cgh.table = "GL", chrom = 5, start = 1561279,
+     end = 16721431, project = "5.p15.33", pvals.to.plot = "pearson.adjp", p.val.thresh = 0.05,
+     cex.label = NULL, heatmap.scale = 2, symbol.width = NULL, plot.symbols = T,
+     main = "5.p15.33-p15.1", device = "PDF", probeID = "symbol")

pdf
  2

> splitHeatmap(basal25expcgh.uSymbol, cgh.table = "GL", chrom = 7, start = 60058297,
+     end = 73190324, project = "7.q11.1", pvals.to.plot = "pearson.adjp", p.val.thresh = 0.05,
+     cex.label = NULL, heatmap.scale = 2, symbol.width = NULL, plot.symbols = T,
+     main = "7.q11.1", device = "PDF", probeID = "symbol")

pdf
  2

> splitHeatmap(basal25expcgh.uSymbol, cgh.table = "GL", chrom = 9, start = 1975288,
+     end = 15136327, project = "9.p24.3-p22.3", pvals.to.plot = "pearson.adjp",
+     p.val.thresh = 0.05, cex.label = NULL, heatmap.scale = 2, symbol.width = NULL,
+     plot.symbols = T, main = "9.p24.3-p22.3", device = "PDF", probeID = "symbol")

pdf
  2

> setwd("..")
> setwd("..")
```

5 Methylation analysis of BCCL

Methylation analysis of BCCL based on Illumina Golden Gate microarray data. Normalise the beta-Values according to the methylumi package, then categorise beta-Values to methylated (if beta-Values are equal or bigger than 0.75), unmethylated (if beta-Values are equal or smaller than 0.25) and partially methylated -all beta-Values inbetween Methylated and unmethylated frequencies and samples are recorded in fData as M.count, PM.count, UM.count and M.samples, PM.samples and UM.samples

```
> dir.create("Methylation_Analysis")
> setwd("Methylation_Analysis")
> basal25met.raw <- readBeadsMethyLumi(paste(filePathStartingFiles, "Basal.25.Methylation.Beadstudio.txt
+     sep = ""))

Reading BeadStudio methylation data

> basal25met.raw <- readPheno(basal25met.raw, paste(filePathStartingFiles, "Basal.25.phenoUpdated2011010
+     sep = ""))

Reading pheno file
25 Pheno sampleNames
25 Eset sampleNames
25 Matching sampleNames
 HCC70 MDAMB468 SUM159 HCC1428 SUM149 HCC1954 MDAMB231 ZR7530 BT549 MDAMB157 HS578T MDAMB436 HCC1143 HCC
Done
```

```
> basal25met <- normalizeMethyLumiBACE(basal25met.raw)
> basal25met <- callStatesMeth(basal25met, thresholds = c(0.25, 0.75))
> validObject(basal25met)
```

```
[1] TRUE
```

Remove CpG island from the autosome chromosome

```
> basal25met <- basal25met[fData(basal25met)$chrom != 23, ]
> unique(fData(basal25met)$chrom)[order(unique(fData(basal25met)$chrom))]
```

```
 [1]  1  2  3  4  5  6  7  8  9 10 11 12 13 14 15 16 17 18 19 20 21 22
```

```
> spitTables(basal25met, "basal25metNoX")
```

```
writing featureData table
Writing phenoData table
Writing betas table
Writing exprs table
Writing flags table
Writing methylated table
Writing mstates table
Writing unmethylated table
Done
```

Count the methylation frequencies for each samples calculated as the fraction of CpG s with value 1

```
> methFrequency <- apply(assayData(basal25met)$mstates, 2, function(x) (sum(x ==
+     1)/nrow(basal25met)))
> methFrequency[order(methFrequency)]
```

```
 [1] 0.2350457 0.2448980 0.2463054 0.2470091 0.2540464 0.2864180 0.2913441 0.3047150 0.3054187
[10] 0.3096411 0.3145672 0.3201970 0.3490500 0.3532723 0.3589022 0.3659395 0.3659395 0.3729768
[19] 0.3771992 0.3793103 0.3821253 0.3884588 0.3905700 0.4264602 0.5073892
```

```
> mean(methFrequency)
```

```
[1] 0.3350880
```

```
> unmethFrequency <- apply(assayData(basal25met)$mstates, 2, function(x) (sum(x ==
+     -1)/nrow(basal25met)))
> unmethFrequency[order(unmethFrequency)]
```

```
 [1] 0.3912738 0.4672766 0.4679803 0.4714989 0.4912034 0.4926108 0.5038705 0.5059817 0.5073892
[10] 0.5179451 0.5249824 0.5285011 0.5390570 0.5446868 0.5601689 0.5643913 0.5707248 0.5876144
[19] 0.5883181 0.6066151 0.6087262 0.6115412 0.6340605 0.6361717 0.6551724
```

```
> mean(unmethFrequency)
```

```
[1] 0.5431105
```

Here we investigated if BCCL cluster according to clinicpath features based on their methylation pattern? First, we removed probes which are only methylated or unmethylated in all samples (these are not informative).

```
> basal25met.var <- basal25met[which(fData(basal25met)$M.count > 1), ]
> basal25met.var <- basal25met[which(fData(basal25met)$UM.count > 1), ]
> length(unique(fData(basal25met.var)$symbol))

[1] 707

> pdf("BCCL25.methylation.pdf")
> sampleDendrogram(basal25met.var, cluster = assayData(basal25met.var)$betas,
+     dist.method = "manhattan", clust.method = "ward", main = "BCCL methylation data")

Cluster method   : ward
Distance         : manhattan
Number of objects: 25

> dev.off()

pdf
  2
```

Overlay Methylation with expression and genomic object, using mapped and flagged probes.

```
> basal25.exp.meth <- expressionMethOverlay(basal25expcgh, basal25met.var, eset.id = "symbol",
+     meth.id = "symbol")

Combining expression and mehtylation ExpressionSets
25 matching sample.ids
1129 probe.ids in common

> validObject(basal25.exp.meth)

[1] TRUE

> length(unique(fData(basal25.exp.meth)$symbol))

[1] 652

> basal25.exp.meth <- expressionMethCorrelation(basal25.exp.meth)

Correlation of gene expression with methylation values

Done
93 significantly correlated genes

Done

> length(unique(fData(basal25.exp.meth)$symbol[fData(basal25.exp.meth)$meth.pearson.adjp <
+     0.05]))

[1] 69

> basal25.exp.meth.uni <- removeReplicateProbesByVariance(basal25.exp.meth)

 652 probes remaining
 Done
```

```
> spitTables(basal25.exp.meth.uni, "basal25.exp.meth.uni")
```

```
writing featureData table
Writing phenoData table
Writing beadNum table
Writing betas table
Writing cgh.exprs table
Writing detection table
Writing exprs table
Writing flags table
Writing GL table
Writing meth.flags table
Writing methylated table
Writing mstates table
Writing se.exprs table
Writing smo table
Writing unmethylated table
Writing GLAD table
Done
```

```
> length(fData(basal25.exp.meth.uni)$symbol[fData(basal25.exp.meth.uni)$meth.pearson.adjp <
+     0.05])
```

```
[1] 55
```

```
> basal25.exp.meth.corr <- basal25.exp.meth[fData(basal25.exp.meth)$meth.pearson.adjp <
+     0.05, ]
```

Investigate if there are any CpGislands differentially methylated in the three groups

```
> basal25exp.meth.c.sam.corr <- dietSAMMulti(basal25.exp.meth.corr, M.table = assayData(basal25.exp.meth
+     pheno = basal25.exp.meth.corr$expression.cluster, fData.ID = "TargetID",
+     fData.genename = "symbol", q.value = 5, logged2 = FALSE, project = "BCCL.meth.SAM.corr",
+     return.eset = T)
```

```
perm= 1
perm= 2
perm= 3
perm= 4
perm= 5
perm= 6
perm= 7
perm= 8
perm= 9
perm= 10
perm= 11
perm= 12
perm= 13
perm= 14
perm= 15
perm= 16
```

```
perm= 17
perm= 18
perm= 19
perm= 20
perm= 21
perm= 22
perm= 23
perm= 24
perm= 25
perm= 26
perm= 27
perm= 28
perm= 29
perm= 30
perm= 31
perm= 32
perm= 33
perm= 34
perm= 35
perm= 36
perm= 37
perm= 38
perm= 39
perm= 40
perm= 41
perm= 42
perm= 43
perm= 44
perm= 45
perm= 46
perm= 47
perm= 48
perm= 49
perm= 50
perm= 51
perm= 52
perm= 53
perm= 54
perm= 55
perm= 56
perm= 57
perm= 58
perm= 59
perm= 60
perm= 61
perm= 62
perm= 63
perm= 64
perm= 65
perm= 66
```

```
perm= 67
perm= 68
perm= 69
perm= 70
perm= 71
perm= 72
perm= 73
perm= 74
perm= 75
perm= 76
perm= 77
perm= 78
perm= 79
perm= 80
perm= 81
perm= 82
perm= 83
perm= 84
perm= 85
perm= 86
perm= 87
perm= 88
perm= 89
perm= 90
perm= 91
perm= 92
perm= 93
perm= 94
perm= 95
perm= 96
perm= 97
perm= 98
perm= 99
perm= 100
1
2
3
4
5
6
7
8
9
10
11
12
13
14
15
16
```

```
17
18
19
20
21
22
23
24
25
26
27
28
29
30
31
32
33
34
35
36
37
38
39
40
41
42
43
44
45
46
47
48
49
50
73 significant probes with local FDR less than %
```

```
> length(unique(fData(basal25exp.meth.c.sam.corr)$symbol))
```

```
[1] 51
```

```
> basal25exp.meth.c.sam.corr.uniq <- removeReplicateProbesByVariance(basal25exp.meth.c.sam.corr)
```

```
 51 probes remaining
 Done
```

```
> spitTables(basal25exp.meth.c.sam.corr.uniq, "basal25exp.meth.c.sam.corr.uniq")
```

```
writing featureData table
Writing phenoData table
Writing beadNum table
Writing betas table
```

```
Writing cgh.exprs table
Writing detection table
Writing exprs table
Writing flags table
Writing GL table
Writing meth.flags table
Writing methylated table
Writing mstates table
Writing se.exprs table
Writing smo table
Writing unmethylated table
Writing GLAD table
Done
```

Make a heatmap of gene expression and methylation and combine them

```
> basal25exp.meth.c.sam.corr.uniq <- basal25exp.meth.c.sam.corr.uniq[, order(basal25exp.meth.c.sam.corr.u
+     decreasing = F)]
> basal25exp.meth.c <- centerBetas(basal25exp.meth.c.sam.corr.uniq)
> meth.sig.atr <- list(NULL)
> meth.sig.atr$order <- 1:ncol(basal25exp.meth.c)
> meth.sig.gtr <- hclust(dist(1 - cor(t(assayData(basal25exp.meth.c)$betas))),
+     method = "ward")
> makeGTR(meth.sig.gtr, project = "BCCL.meth.51.exp.order")
> makeCDT(meth.sig.gtr, meth.sig.atr, basal25exp.meth.c, Mtable = assayData(basal25exp.meth.c)$betas,
+     project = "BCCL.meth.51.exp.order")
> basal25exp.meth.c <- centerGenes(basal25exp.meth.c, center = "mean")
> makeGTR(meth.sig.gtr, project = "BCCL.meth.51.exp.order.gex")
> makeCDT(meth.sig.gtr, meth.sig.atr, basal25exp.meth.c, Mtable = exprs(basal25exp.meth.c),
+     project = "BCCL.meth.51.gex.Mean")
> setwd("..")
```
